# High divergence in primate-specific duplicated regions: Human and chimpanzee *Chorionic Gonadotropin Beta *genes

**DOI:** 10.1186/1471-2148-8-195

**Published:** 2008-07-07

**Authors:** Pille Hallast, Janna Saarela, Aarno Palotie, Maris Laan

**Affiliations:** 1 Department of Biotechnology, Institute of Molecular and Cell Biology, University of Tartu, Riia 23, 51010 Tartu, Estonia; 2 Department of Molecular Medicine, National Public Health Institute, Haartmaninkatu 8, 00290 Helsinki, Finland; 3 Finnish Genome Center, Biomedicum Helsinki, University of Helsinki, Haartmaninkatu 8, 00290 Helsinki, Finland; 4 The Broad Institute of Harvard and MIT, Cambridge Center, Cambridge, MA 02142, USA; 5 Wellcome Trust Sanger Institute, Hinxton, Cambridge, CB10 1SA, UK

## Abstract

**Background:**

Low nucleotide divergence between human and chimpanzee does not sufficiently explain the species-specific morphological, physiological and behavioral traits. As gene duplication is a major prerequisite for the emergence of new genes and novel biological processes, comparative studies of human and chimpanzee duplicated genes may assist in understanding the mechanisms behind primate evolution. We addressed the divergence between human and chimpanzee duplicated genomic regions by using Luteinizing Hormone Beta (*LHB*)/Chorionic Gonadotropin Beta (*CGB*) gene cluster as a model. The placental *CGB *genes that are essential for implantation have evolved from an ancestral pituitary *LHB *gene by duplications in the primate lineage.

**Results:**

We shotgun sequenced and compared the human (45,165 bp) and chimpanzee (39,876 bp) *LHB/CGB *regions and hereby present evidence for structural variation resulting in discordant number of *CGB *genes (6 in human, 5 in chimpanzee). The scenario of species-specific parallel duplications was supported (i) as the most parsimonious solution requiring the least rearrangement events to explain the interspecies structural differences; (ii) by the phylogenetic trees constructed with fragments of intergenic regions; (iii) by the sequence similarity calculations. Across the orthologous regions of *LHB/CGB *cluster, substitutions and indels contributed approximately equally to the interspecies divergence and the distribution of nucleotide identity was correlated with the regional repeat content. Intraspecies gene conversion may have shaped the *LHB/CGB *gene cluster. The substitution divergence (1.8–2.59%) exceeded two-three fold the estimates for single-copy loci and the fraction of transversional mutations was increased compared to the unique sequences (43% versus ~30%). Despite the high sequence identity among *LHB/CGB *genes, there are signs of functional differentiation among the gene copies. Estimates for d_n_/d_s _rate ratio suggested a purifying selection on *LHB *and *CGB8*, and a positive evolution of *CGB1*.

**Conclusion:**

If generalized, our data suggests that in addition to species-specific deletions and duplications, parallel duplication events may have contributed to genetic differences separating humans from their closest relatives. Compared to unique genomic segments, duplicated regions are characterized by high divergence promoted by intraspecies gene conversion and species-specific chromosomal rearrangements, including the alterations in gene copy number.

## Background

Gene duplication has long been considered as one of the main mechanisms of the adaptive evolution and as an important source of the genetic novelty [1]. Differential duplications and deletions of chromosomal regions including coding genes provide a powerful source for the evolution of species-specific biological differences [2]. Compared to other mammals, the genomes of primates show an enrichment of large segmental duplications with high levels (>90%) of sequence identity [3]. In the human genome particularly pronounced expansions of the copy number have been reported for genes involved in the structure and function of the brain [4]. In comparison of human and its closest relative chimpanzee, large duplications contribute considerably (2.7%; [5]) to the overall divergence compared to single base pair substitutions (1.2–1.5%; [2,6-12]). In addition to providing the substrate for non-allelic homologous recombination mediating genomic disorders (reviewed by [13]), the duplication architecture of a genome may also influence normal phenotypic variation. It has been estimated that ~20% of segmental duplications are polymorphic within human and chimpanzee populations contributing to intraspecies diversity [5,14]. Despite the fact that segmental duplications cover a substantial fraction of the great apes genomes, the experimental data on the divergence and detailed evolutionary dynamics of duplicated gene regions is still limited. Sequence comparison of duplicated genes in sister-species would assist in understanding the mechanisms behind primate evolution and in associating the genetic divergence with phenotypic diversification.

**Figure 1:**
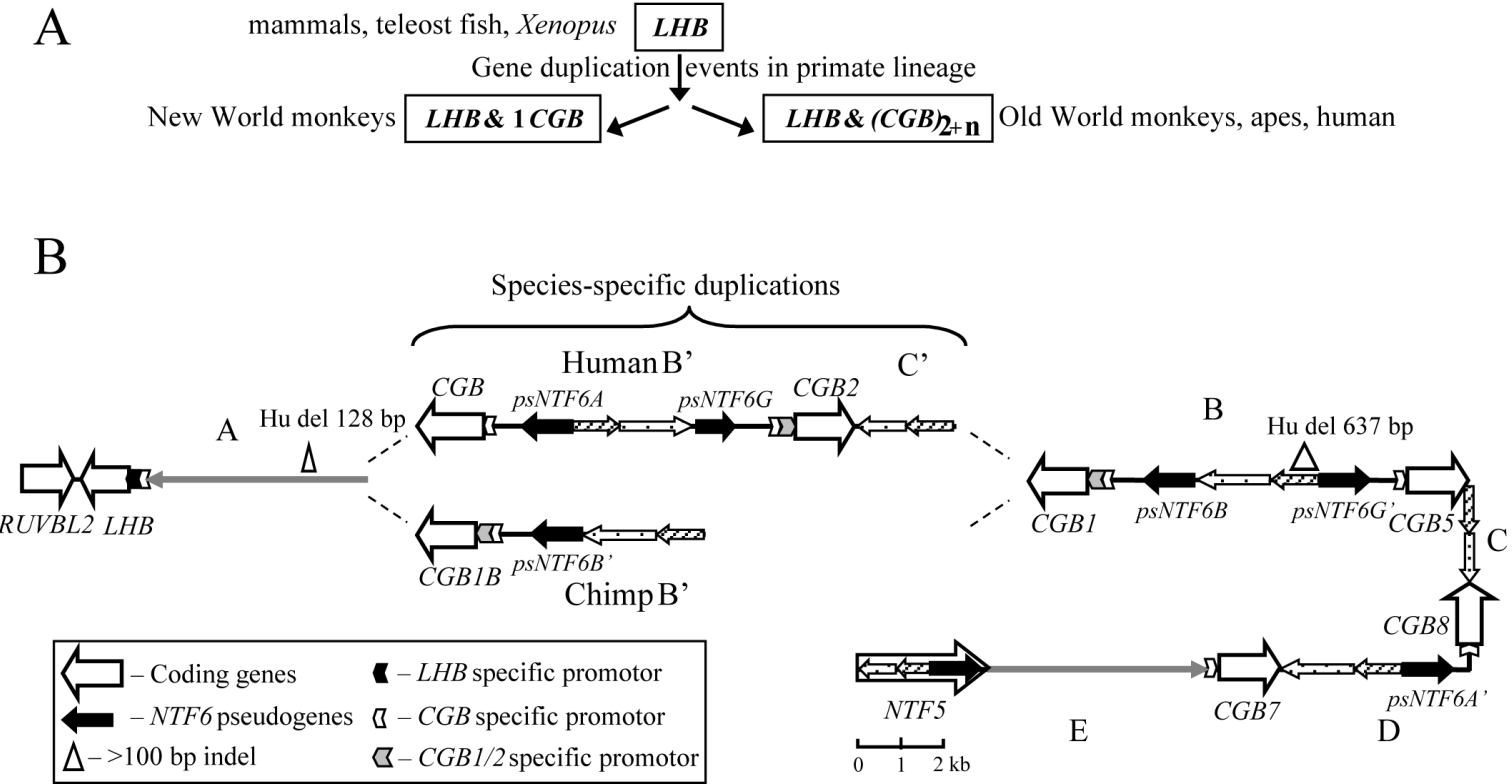
**Evolution of the *Gonadotropin Hormone Beta *(*CGB*) genes**. Duplication of ancestral *Luteinizing Hormone Beta *(*LHB*) gene in primate lineage has given rise to a novel gene, *CGB*. (A) A simplified schematic presentation of the evolution of *LHB/CGB *genes in primates [15,16]. (B) Comparative structure of the human (GenBank reference: NG_000019) and the chimpanzee *LHB/CGB *cluster (this study, Genbank: accession number EU000308) drawn to an approximate scale. Coding genes are depicted as wide empty arrows in the direction of transcription on the sense strand. *A *– *E *indicate the *intergenic regions*. *B' *and *C' *denote putative duplications of the *intergenic regions B *and *C*. Identical color and pattern codes refer to the DNA segments within the cluster with highly similar sequences, the direction of the DNA sequence is indicated on the sense strand. Sequence identity within the cluster: between coding genes 85–99%; *intergenic regions A *and *E *81%; *C *and *C' *96%; *B*, *B' *and *D *ranging 81–98%.

One of the genomic regions that has evolved through several gene duplication events in primate lineage is the Luteinizing Hormone Beta (*LHB*)/Chorionic Gonadotropin Beta (*CGB*) gene cluster locating in human at 19q13.32. The *LHB/CGB *genes have an essential role in reproduction: placentally expressed HCG hormone contributes to the implantation process of the embryo during the early stages of pregnancy, pituitary expressed luteinizing hormone promotes the ovulation and luteinization of follicles and stimulates the steroidogenesis. In human, the cluster consists of seven highly homologous genes: an ancestral *LHB *and six duplicated *CGB *genes [15]. The data from other primates supports the hypothesis of several sequential duplication events increasing gradually the number of *CGB *genes among primates from one (New World monkeys: the owl monkey, *Aotus trivirgatus *and the dusky titi monkey, *Callicebus moloch*) to six in human (Figure 1A). The mapped copy number of the *CGB *gene among Old World monkeys varies: three in rhesus macaque (*Macaca mulatta*), five in guereza monkey (*Colobus guereza*) and dusky leaf monkey (*Presbytis obscura*), four in orangutan (*Pongo pygmaeus*) [16]. It has been suggested that *CGB *gene first arose in the common ancestor of the anthropoid primates after diverging from tarsiers [16].

We have chosen *LHB/CGB *genomic region as a model to study the evolution of recent primate duplications. Although the chimpanzee genome has been sequenced, there are still large gaps and uncertainties concerning the segmental duplications regions, including the *LHB/CGB *region. To obtain a high-quality DNA sequence, we constructed and sequenced a shot-gun library, and hereby report the complete sequence of the entire *LHB/CGB *cluster in the common chimpanzee. We are addressing the following aspects regarding to the evolution of duplicated genes in closely related species: (i) in-depth comparison of the human and chimpanzee *LHB/CGB *genome clusters; (ii) variation in substitution rates; (iii) genic and intergenic divergences; (iv) impact of intraspecies gene conversion in phylogeny, divergence and transversion/transition ratios; (v) evidence of natural selection. To our knowledge, this is the first detailed report of parallel independent duplication events initiated within a duplicated genome cluster and leading to structural divergence in the two sister-species, human and common chimpanzee.

## Results and Discussion

### Human and chimpanzee *LHB/CGB *genome clusters differ considerably in size

The total length of the sequenced chimpanzee (*Ch*) *LHB/CGB *genomic region obtained from two overlapping BAC clones was 43,945 bp. It encompasses 1,029 bp of the flanking (centromeric side) *RUVBL2 *gene, 39,876 bp of the entire *LHB/CGB *cluster and 2,986 bp of the flanking (telomeric side) *NTF5 *gene (Figure 1B; Genbank submission: EU000308). Compared to the human (*Hu) LHB/CGB *region (Genbank: NG_000019), the sequence of the *ChLHB/CGB *cluster is 5,289 bp shorter. The sequence characteristics of the *LHB/CGB *genomic regions are similar in these two species: extremely high GC-nucleotide content (57% compared to average 41% for *Hu *and *Ch *[10]), high fraction of CpG islands (*Hu *6.6%, *Ch *6.1% compared to estimated 1–3.5% for *Hu *and *Ch *[6,8]) and repetitive sequences (*Hu *26.9%, *Ch *25.15%), especially SINEs (*Hu *23.23%, *Ch *21.81%) (Additional file 1). High repeat content is also characteristic to several other duplicated regions, such as *MHC *class I region [7] and *Apolipoprotein CI *genomic segment [17].

As expected, there is a considerable similarity between the genomic organization of human and chimpanzee *LHB/CGB *clusters (Figure 1B). We identified two highly identical, apparently orthologous segments within the cluster: *RUVBL2/LHB/intergenic region A *(Ch 8,084 bp, Hu 7,973 bp; 96% sequence identity) and the region spanning from *CGB1 *to *NTF5 *(Ch 29,136 bp, Hu 28,568 bp; 94.8% sequence identity). However, a large species-specific structural rearrangement was localized between the *intergenic region A *and *CGB1 *gene, resulting in discordant size of human (45,165 bp) and chimpanzee (39,876 bp) clusters as well as species-specific number of duplicated gene copies, seven for human (1 *LHB *+ 6 *CGB *genes) and six (1 *LHB *+ 5 *CGB*) for chimpanzee. In human the rearranged region (12,700 bp) harbors one HCG beta coding *CGB *gene and one *CGB1/2*-like gene (*CGB2*) recognized by a specific promoter-segment [18,19], while in chimp (rearranged region 6,725 bp) only a *CGB1/2*-like gene (*CGB1B*) is present in an inverted orientation compared to human. In addition, *ChLHB/CGB *cluster lacks the whole *intergenic region C' *and has a considerably shorter inverted *intergenic region B' *(Figure 1B).

### Evidence for independent duplication events within *LHB/CGB *gene cluster for two closely related primate species

**Figure 2:**
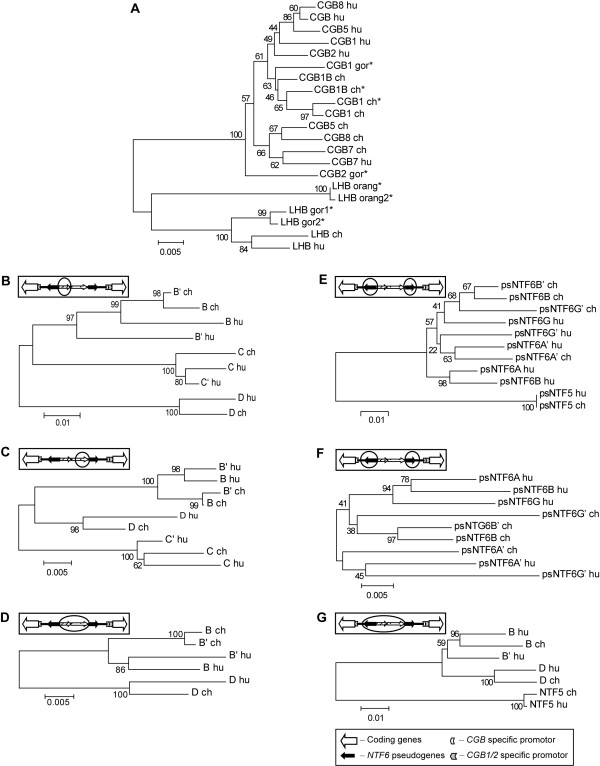
**Neighbor-joining trees based on genic (A) and intergenic (B-G) regions within *LHB/CGB *gene cluster**. (A) A phylogenetic tree of the full sequences of *LHB/CGB *gene from *Homo sapiens*, *Pan troglodytes*, *Gorilla gorilla *and *Pongo pygmeaus*. (*) denotes sequences from [44]. Phylogenetic analysis of intergenic regions was conducted with segments without (B-D) and with (E-G) covering *NTF6 *pseudogenes. The homologous segments used for each respective phylogenetic analysis are indicated with a circle on a consensus structure of the intergenic regions in *LHB/CGB *cluster (boxed; from Figure 1B). The nomenclature of the intergenic regions is as on Figure 1B. Bootstrap support values are shown at the nodes (1000 bootstrap replications). Abbreviations: hu – human, ch – chimpanzee, gor – gorilla, orang – orangutan.

We considered alternative scenarios that may have led to structural differences in *LHB/CGB *clusters in two sister-species. The scenario of species-specific parallel duplications was supported by several lines of evidence (Figure 1B, Figure 2). First, it was the most parsimonious solution requiring the smallest number of rearrangement events. Assuming that the ancestral *Hu-ChLHB/CGB *cluster consisted of the present-day highly identical segments (Figure 1B; see above), only one evolutionary event would explain the current structure of *ChLHB/CGB *cluster – a direct duplication of *CGB1 *and most of *intergenic region B *(excluding *psNTF6G'*) giving arise to the segment *CGB1B/Ch-B'*. In humans, two events would have lead to present *HuLHB/CGB *(not in order) – an inverted duplication of the entire region from *CGB1 *to *CGB5 *gene (segment from *CGB *to *CGB2*) and a direct duplication and translocation of *region C *creating *C' *next to *CGB1 *gene (Figure 1B). These parallel duplications in two sister-species might have been initiated by non-allelic homologous recombinations between multiple *Alu *SINE sequences (*Alu*Sx, *Alu*Sp, *Alu*Sq) locating at the junctions of both human- and chimp-specific duplications. Consistently, the phylogenetic trees that were constructed using different fragments of intergenic regions show that *Ch-B *and *Ch-B'*, *Hu-B *and *Hu-B' *as well as *Hu-C *and *Hu-C' *clearly cluster together supporting their paralogous status (Figure 2B–F). The *Hu-D *and *Ch-D *region form a well-supported clade (Figure 2B–D, G) giving evidence that these are orthologous segments. The phylogenetic relationship supports the ancestral status of *region C *(present in both species) compared to *Hu-C' *(human-specific duplication). Also the sequence similarity calculations are consistent with the scenario of independent species-specific duplications: the identity of *Ch-B *and *Ch-B' *regions is 98.1%, and of *ChCGB1 *and *ChCGB1B *98.7%. The latter exceeds the estimates for any orthologous genes between human and chimp in *LHB/CGB *cluster (from 98.2% in *LHB *to 97.4% in *CGB5 *and *CGB8*). However, the possibility of more intensive gene conversion between these intergenic segments resulting in higher intraspecies similarity cannot be excluded. It is well known that past intraspecies gene conversion events might be reflected by tree phylogenies and could lead to erroneous conclusions [16,20]. The footprint of gene conversion is also reflected on the phylogenic tree of human and chimpanzee *LHB/CGB *genes (Figure 2A). Instead of two separate clades for orthologous *CGB5 *and *CGB8*, the genes within one species cluster together.

Alternative scenarios leading to discordant gene number in *LHB/CGB *gene clusters in these two species are less supported. A minimum of three rearrangement events (Figure 1B) would have been required for the chimpanzee-specific deletion: loss of *CGB *and *psNTF6A *gene accompanied with the inversion of *CGB2 *and *intergenic region B' *(giving rise to *CGB1B *and chimp *B'*), and a separate deletion of *region C' *in chimp. Also the scenario of human-specific duplication (Figure 1B) would have required at least three events: an inversion of *CGB1B *and *intergenic region Ch-B' *(giving rise to part of *Hu-B' *and *CGB2*); either a direct duplication and translocation of *CGB8 *gene along with *psNTF6A' *or an inverted duplication and translocation of *CGB5 *along with *psNTF6G' *(resulting in *CGB *and *psNTF6A*), and a direct duplication and translocation of *intergenic region C *creating *Hu-C' *next to *CGB1 *gene.

A number of gene families have been characterized where the gene number differs between human and chimpanzee due to species-specific indels [7,21]. To our knowledge, this is the first report where parallel independent duplications arisen within the same region in human and chimpanzee genomes give the best explanation for the observed structural differences between two sister-species. However, there are examples of independent duplications among primates resulting in convergent functions. A more recent duplication of *X-linked opsin *gene in New World howler monkeys (*Alouatta seniculus *and *Alouatta caraya*) compared to Old World primates, has lead to full trichromacy [22-24] and also there are independently arisen functionally close genes within the *Growth Hormone/Somatomammotropin *genome cluster in New World monkeys and Old World monkeys/hominoid lineages [20,25].

### Comparative nucleotide divergence profiling of the orthologous regions reveals non-uniform substitution rates across the *LHB/CGB *region

**Figure 3:**
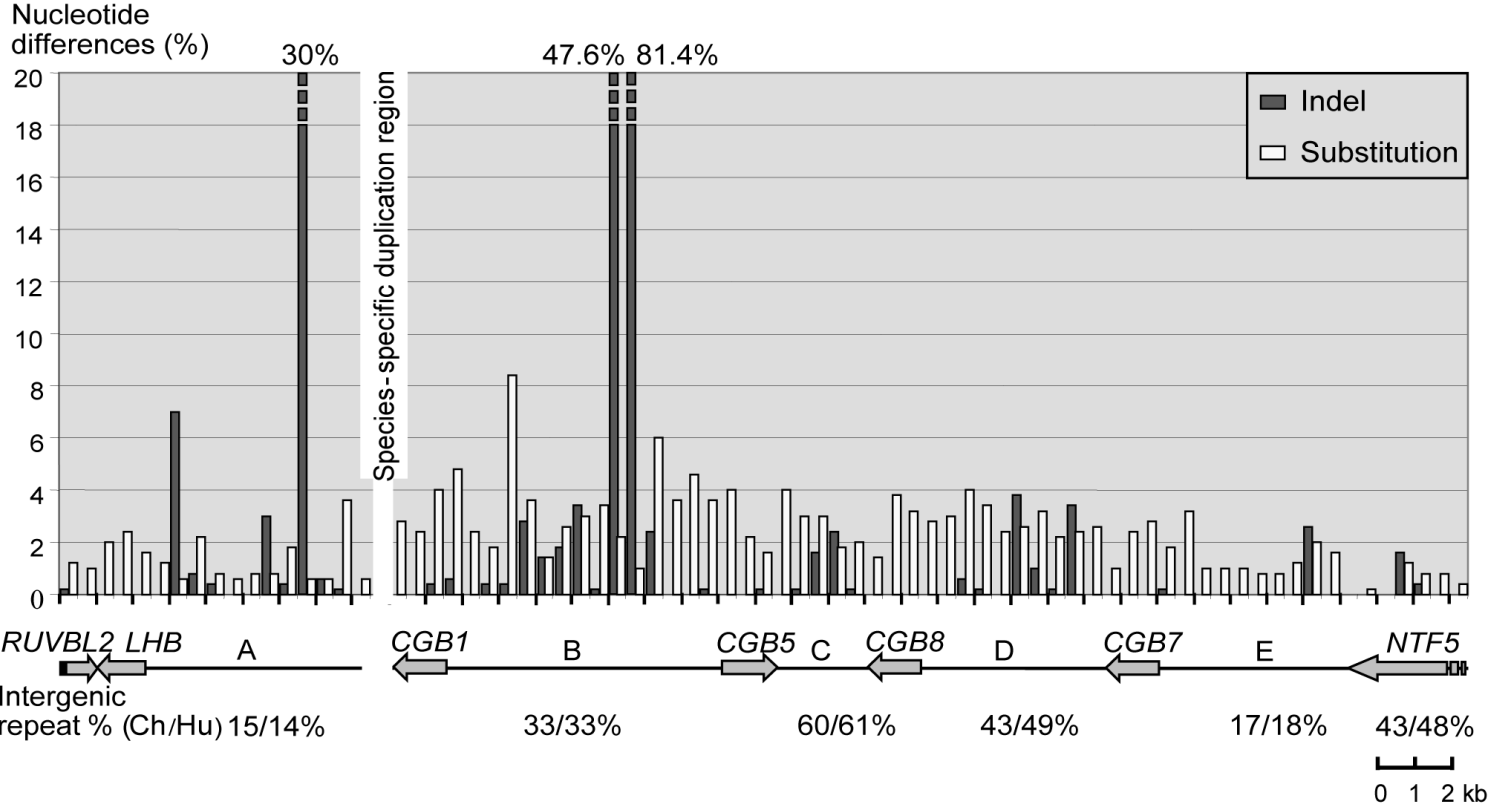
**Divergence profile between orthologous regions of the human and the chimpanzee *LHB/CGB *clusters**. In total the compared region covered 37,220 bp (Ch)/36,541 (Hu), including 8,084 bp (Ch)/7,973 bp (Hu) from *RUVBL2 *gene to the end of *intergenic region A *and 29,136 bp (Ch)/28,568 bp (Hu) from *CGB1 *to *NTF5 *gene. The species-specific large duplications (human 12,700 bp, chimp 6,725 bp) have been excluded from the comparison. The percents of nucleotide substitutions and indels are calculated per 500 bp non-overlapping windows. Grey arrows indicate the locations of coding genes drawn to an approximate scale. *A *– *E *denote intergenic regions from Figure 1B. Intergenic repeat fraction includes *SINEs, LINEs*, satellites, simple repeats and low complexity DNA sequences within each intergenic region.

We generated a comparative nucleotide divergence profile of substitutions and indels across the orthologous genomic regions of *LHB/CGB *cluster (Figure 1B, Figure 3). Human (36,541 bp) and chimpanzee (37,220 bp) aligned genomic sequences were analyzed using a non-overlapping sliding window of 500 bp. Several studies have suggested that the majority of the genomic divergence between human and chimpanzee comprises of indels (3.0–11.9%) compared to contribution of nucleotide substitutions (1.2–1.5%) [2,6-11,26]. In the duplicated *LHB/CGB *region indels (2.7%) and substitutions (2.3%) contributed approximately equally to the total divergence (5%) between the two species. In total, 61 indels (mean 16; range 1–637 bp) were identified, 26 as human and 35 as chimpanzee deletions. The size distribution of these indels was consistent with previous reports [2,6,9-11] revealing an excess of short indels: 44% involved a single basepair, 77% 1–5 bp and 96.7% <100 bp (Additional files 1 and 2). Two large indels of >100 bp co-locate with repetitive elements: the 128 bp long indel in *intergenic region A *is located in a simple-repeat rich region; the 637 bp indel in *intergenic region B *is flanked and composed of SINE sequences (Figure 1B, Figure 3). The latter indel could be defined as a recent human-specific sequence loss since the duplicated human-specific *intergenic region B' *lacks this deletion. High proportion (>50%) of indels identified between human and chimpanzee has been shown to contain repetitive elements [6,7]. The regional content of repeats was also correlated with the non-uniform distribution of the nucleotide identity across the cluster (Figure 3; Additional file 1) consistent with the data that repetitive sequences, e.g. *Alu *repeats have a higher rate of base substitutions [27].

### Transition to transversion ratio in duplicated regions differs from the estimations for unique genomic sequences: possible role of gene conversion

**Figure 4:**
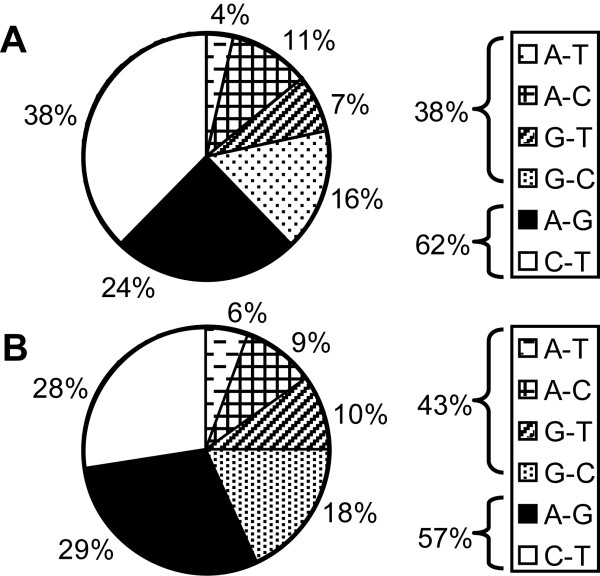
**Profile of nucleotide substitutions in human and chimpanzee orthologous *LHB/CGB *genes**. Grouping of nucleotide substitutions: (A) Nucleotide substitutions in orthologous *LHB*, *CGB1, CGB5, CGB8 *and *CGB7 *genes (in total 6,878 bp; GC-nucleotide content 64%; 161 substitutions). (B) Nucleotide substitutions in the whole orthologous region of the *LHB/CGB *genome cluster (in total 36,211 bp, GC-nucleotide content 57%, 835 substitutions). Percents for all substitution types are shown with summarized information for transversions and transitions.

We investigated the distribution of nucleotide substitutions within orthologous regions of *LHB/CGB *cluster in more detail (Figure 4). Transitions (C⇔T, A⇔G) and transversions (T, C⇔A, G) were found to contribute 62% and 38% of the total substitutions in five orthologous *LHB/CGB *genes, respectively (Figure 4A). The corresponding estimates for the whole orthologous region (including genes) were 57% for transitions and 43% for transversions (Figure 4B). Notably, the contribution of transitions is ~10% lower than reported in previous studies comparing human and chimpanzee genomic regions (68.87% – 70.3%) [7,8]. The most frequent transversions are G⇔C substitutions, contributing 16% of all substitutions in genes and 18% across entire orthologous region, exceeding previous estimations by twofold (9.14%, 9%) [7,8]. Consistently, a large excess of G⇔C transversional pairs as compared to other substitutions has been reported for human *HSP70 *and mouse *Hsp70 *orthologous duplicate genes [28]. The high proportion of transversions in the *LHB/CGB *cluster can be explained by biased gene conversion [29] that leads to a high GC content (57% in human and chimp *LHB/CGB *region) and thus increases the probability of G⇔C substitutions due to the altered base composition [28,30,31].

Among the transitions, we observed an excess of C⇔T substitutions in *LHB/CGB *genes (38%) versus the whole region (28%). It is generally accepted that a high proportion of transitions are C to T substitutions in CpG dinucleotides, exhibiting about 10 times higher mutation rate than the genomic average [9,32]. A higher GC content (64% vs 57%) and presence of CpG islands could explain an excess of C⇔T substitutions in *LHB/CGB *genes compared to intergenic regions.

### Sequence divergence between human and chimpanzee duplicated *LHB/CGB *genes is higher than estimates for single copy genes

**Figure 5:**
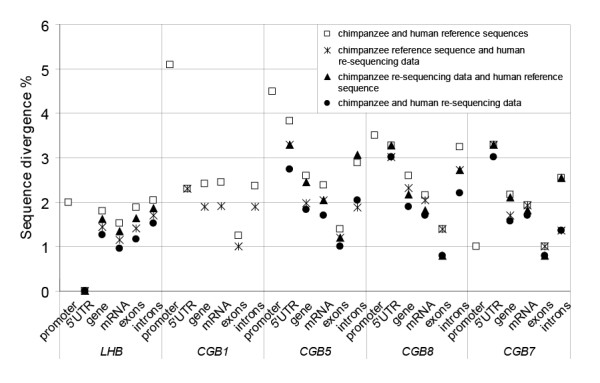
**Nucleotide divergence (%) between human and chimpanzee orthologous *LHB *and *CGB *genes calculated for promoter, 5'UTR, genic, mRNA, exonic and intronic regions.** Divergence was estimated by using the human (GenBank: NG_000019) and chimpanzee (this study, Genbank: EU000308) reference sequences alone or by incorporating the diversity data obtained from re-sequencing for one or both species into the calculations. The re-sequencing data for human (n = 95) originated from the published study[29] and for chimpanzee (n = 11) from the unpublished dataset of the authors.

We studied the human and the chimpanzee orthologous genes *LHB*, *CGB1*, *CGB5*, *CGB8 *and *CGB7 *for nucleotide divergence. The nucleotide divergence ranged from 1.8% for *LHB *to 2.59% for *CGB5 *and *CGB8 *genomic sequences (Figure 5; Additional file 3). Despite that the coding regions are most conserved among the species, the exonic divergence rates (mean 1.39%; range 1 – 1.88%) exceeded many times the previous estimates for single-copy regions [8-10,12,26,33]. In non-coding regions, the average nucleotide divergence for promoter regions was as high as 3.22% (range 1 – 5.1%), for introns 2.62% (range 2.04 – 3.24%) and for 5' UTR 2.54% (range 0 – 3.83%). The comparative estimates for single-copy genes are much lower: in promoters 0.75–0.88%, in exons 0.51–1.09%, in introns 1.03–1.47% and in 5' UTR 1.00–1.41% [10-12,26,33-36]. It has been suggested that interaction of selection and gene conversion contributes to a higher divergence and diversity in multigene families compared to single-copy genes [37,38]. As any *de novo *mutation has a potential to be spread by gene conversion from the original locus to other gene copies, every duplicate could accumulate substitutions arisen in neighboring genes.

Notably, when the polymorphism data from re-sequencing studies of the human (n = 95 individuals) [29] and the chimpanzee (n = 11 individuals) [Hallast et al, unpublished] *LHB/CGB *genes were incorporated into calculations, the sequence divergence in genes dropped from 1.8% to 1.26% in *LHB*, from 2.59% to 1.84% in *CGB5*, from 2.59% to 1.9% in *CGB8 *and from 2.18% to 1.57% in *CGB7 *(Figure 5; Additional file 3). Still, a higher divergence compared to the published data for single-copy genes remained.

Most importantly, our data indicates that the divergence estimates between human and chimpanzee might be substantially lower than reported when the intraspecies variation is taken into account.

### Duplicated, highly homologous *LHB/CGB *genes evolve under different selective constraints

A number of non-synonymous changes were identified in human-chimpanzee comparison: two in *LHB*, two in *CGB8*, three in *CGB5 *and *CGB7 *and four in *CGB1 *(Additional file 3). None of the differences found in exonic regions in chimp altered the ORF nor created a preliminary stop-codon compared to human genes. The maximum likelihood (ML) method was used to estimate ω, non-synonymous (d_n_)/synonymous (d_s_) rate ratio for the orthologous genes (n = 5) in the human and chimpanzee *LHB/CGB *gene cluster (Table 1) [39]. For four of the compared genes (*LHB, CGB5, CGB8, CGB7*) the parameter was ω < 1, indicating purifying selection. An especially low ω estimate was obtained for *LHB *(ω = 0.087; 6 synonymous, 2 non-synonymous changes; amino acid divergence 1.42%) and *CGB8 *(ω = 0.099; 5 synonymous, 2 non-synonymous changes; amino acid divergence 1.21%). Consistently, the d_n_/d_s_ratio calculated by an alternative Li93 method [40,41] was statistically significant (d_n_/d_s _= 0.146; p = 0.049 two-tailed Z-test [42]) for *LHB *and reached borderline significance for the *CGB8 *(d_n_/d_s _= 0.162; p = 0.078) (Additional file 4). Indeed, the *LHB *gene is highly conserved throughout vertebrate evolution in association with its function – it is coding for the receptor-binding beta subunit of luteinizing hormone (LH) critical for successful reproduction [15]. The functional constraint of the human *CGB8 *may be associated with its major role in contributing to the *HCG beta *subunit mRNA transcript pool compared to other *CGB *genes [43].

**Table 1:** Maximum likelihood estimation of ω (= d_n_/d_s_) values by PAML analysis and amino acid divergence in human and chimpanzee orthologous genes.

Gene	n^a^	ω^b^	AA %^c^
LHB	141	0.087	1.42
*CGB1*	132	2.658	3.03
*CGB5*	165	0.180	1.82
*CGB8*	165	0.099	1.21
*CGB7*	165	0.378	1.82

^a ^number of codons in the sequence

^b ^non-synonymous/synonymous rate ratio, averaged over sites (d_n_/d_s_).

^c ^Divergence at amino acid level (%).

*CGB1 *gene (ω = 2.658, 1 synonymous, 4 non-synonymous changes; amino acid divergence 3.03%) stood out as the only locus in the gene cluster with estimated ω >1, which would be consistent with positive or adaptive evolution. It has been suggested that *CGB1 *has arisen in the common ancestor of African great apes through a duplication event accompanied by an insertion of a novel putative promoter, 5'UTR and exon 1. So far, the detection of *CGB1 *gene has been unsuccessful in orangutan *Pongo pygmaeus *by using the PCR approach [44] and in macaque *Macaca mulatta *(Genbank: AC202849) by *in silico *search of the current genome assembly. It has been shown that in human the contributions of *CGB1 *and its duplicate human-specific *CGB2 *to the summarized expression of the six *CGB *genes in placenta is much lower (1/1000 to 1/10000) compared to their gene dosage (two genes out of six total) [43,45]. However, in testis the proportional contribution of *CGB1/2 *to the total *CGB *transcript pool is as high as 1/3 [45], which may indicate a possible role of these genes in male reproductive tract. Indeed, a recent study has shown that HCG alpha and HCG beta free subunits are produced in high amounts in the prostate and testes and are subsequently observed in seminal plasma [46].

In order to address which parts of the studied genes exhibit signals of evolving under positive selection, we used CRANN analysis calculating d_n _and d_s _values for sliding and overlapping windows along individual genes (Additional file 5) [47,48]. In case of *HCG beta *subunit coding genes (*CGB5*, *CGB8 *and *CGB7*), the patterns of nucleotide differences between the sister species were similar – across the protein the synonymous substitutions exceeded the non-synonymous ones and were concentrated in the N- and C-terminus of the protein. In contrast, in *CGB1 *the non-synonymous substitutions dominated and were distributed in the signal peptide (amino acids 1–20) and the centre of the protein.

### Impact of gene duplications in primate divergence

Primate-specific gene duplications have involved loci regulating immunity (e.g. *MHC, beta-defensin*, *CD33rSiglec *gene clusters), reproduction (e.g. *LHB/CGB*, *GH/CSH*, *PRAME genes; Y-chromosomal gene families*), development and adaptation (e.g. *Beta Globin, Opsin*, *Rh blood group*, *Class 1 ADH*, *PRDM *and *FAM90A *gene families), and brain functions (e.g. *NAIP, ROCK1, USP10 and MGC8902 *genes) [4,16,20,22,49-59]. It has been suggested that these duplication events may have been facilitated by non-allelic homologous recombination between *Alu *sequences, expanded into millions of copies all over primate genomes [54,60]. There are several examples of independent duplication events in the New World monkey (NWM) and Old World monkey (OWM)/hominoid lineages as well as in distinct primate species. For example, the OWM and apes have three *Opsin *genes and are trichromats due to gene duplication at the base of the OWM lineage. In NWM, the situation is more variable: most species exhibit two *Opsin *genes, but in the howler monkey an additional gene duplication has led to full trichromacy [23,24]. In *Growth Hormone *gene cluster (five to eight gene copies) some of the duplicate genes in the OWM/hominoid lineages have acquired a novel function and code for Chorionic Somatomammotropin (*CSH *genes) involved in the glucose metabolism of the fetus and the mother. However, the CSH genes are missing from the genomes of NW monkeys [61]. Further species-specific duplications of *GH/CSH *genes have been reported for gibbon, macaque, chimpanzee and human [62,63]. In human-chimpanzee comparison, only three *GH/CSH *genes are clearly orthologous [21]. Other gene clusters with independent gene duplications in human, apes and macaque lineages are *MHC *and testes-expressed *PRAME *genes [53,64]. The ancestral *MHC-B *duplicated into *MHC-B *and *MHC-C *in hominoids. While human *MHC-C *orthologs are found in African apes and orangutans, they are not present in macaque or any other OW monkeys [65,66]. Species-specific evolutionary scenarios have also been reported for *LHB/CGB *genes [16]. In addition to the structural differences between human and chimpanzee *LHB/CGB genes *reported in this study, an expansion of *CGB *genes up to 50 gene copies has been shown in gorilla (*Gorilla gorilla*) [4,49]. Despite the high number of structural differences that has been shown between human and chimpanzee genomes by *in silico *whole-genome analysis (reviewed in [67]), the experimental data for copy number differences between these sister-species has been reported only for a few gene clusters (e.g. *GH/CSH, LHB/CGB, PRAME, MGC8902, CXYorf1, KGF, CD33rSiglec, NANOG*) [16,20,52,53,59,68-70].

In addition to creating structural divergence among the species, duplications provide also the bases for diversification of gene functions. For primate-specific gene duplications, there is evidence of variability in evolutionary rates among the gene copies within and among the species, and of different selective constraints acting on different members of the gene clusters, such as *MHC, beta-globin*, *GH/CSH, PRAME*, *Rh blood group*, *CD33rSiglec *and *beta-defensin *genes [71-73]. For example, in human-chimpanzee comparison, the chimp *MHC *class I loci *A, B *and *C *are characterized by lower intra-species allelic variation compared to human, providing evidence that ancestral chimpanzee populations may have experienced a selective sweep [74,75]. In marmoset *LHB/CGB *region, a switch of functions has happened between the ancestral *LHB *and the derived *CGB *gene. Although *LHB *and *CGB *genes are both present at the genomic level, only *CGB *gene is expressed in the pituitary and placenta tissues. Thus, Chorionic Gonadotropin Hormone is the only gonadotropin carrying also the luteinizing function fulfilled by Luteinizing Hormone in mammals [76,77].

Duplicated genes tend to evolve *in consort *facilitated by active inter-locus gene conversion increasing and preserving sequence similarity among the gene copies [37,38,78]. Concerted evolution within species may lead to erroneous phylogenetic trees and to the overestimation of interspecies divergence dates [20,79,80]. Incorporation of gene conversion data into the equations for calculating interspecies divergence may be still a challenge requiring detailed knowledge of a particular genomic region.

So far, only a limited number of reports has been published that focus on detailed variation patterns of duplicated gene families in primates (*MHC*, *Globin*, *GH/CSH *and *LHB/CGB *genes) [7,29,81-83]. However, the common observation is that compared to single-copy segments, duplicated regions tend to exhibit higher interspecies diversity that could be explained by relaxed selective pressures and/or gene conversions spreading mutations. Thus, when calculating the divergence in duplicated primate-specific regions, the inclusion or exclusion of intraspecies variation data into equations may have a substantial impact on the divergence estimates.

## Conclusion

We compared the human and chimpanzee duplicated *LHB/CGB *genome clusters and hereby present the detailed evidence for parallel independent duplication events in the two sister-species resulting in discordant number of *CGB *genes (6 in human, 5 in chimpanzee). To our knowledge, this is the first detailed report of parallel duplications in these sister-species leading to structural divergence. The evolutionary fate of duplicated genes is shaped by the interaction of gene conversion and selection. In *LHB/CGB *gene cluster, active gene conversion may have contributed to higher interspecies sequence divergence (both genic and intergenic sequences) and altered transition/transversion ratio compared to the single-copy loci. This higher divergence remained when intraspecies variation was taken into account. However, the drop in divergence estimates after incorporating the intraspecies variation data promotes to reanalyze previously studied loci, where the human-chimpanzee divergence may be substantially lower than initially calculated. Despite the high sequence homology among *LHB/CGB *genes (85–99%), there are signs of functional differentiation among the gene copies. To reconstruct the full evolutionary history of *LHB/CGB *gene cluster, further studies are required comprising high-quality sequence data from several primate species.

## Methods

### Bacterial Artificial Chromosome (BAC) screening and shotgun library construction

BAC library of common chimpanzee (*Pan troglodytes*) RPCI-43 was obtained from BACPAC Resource Center at the Children's Hospital Oakland Research Institute (Oakland, CA). In order to identify BAC clones containing *LHB/CGB *genome cluster we used recommended protocols and performed hybridization screening with a PCR-product containing *LHB*-specific sequence amplified from chimpanzee genomic DNA. Probe DNA was labeled with [^32^P]dCTP by random primer extension using DecaLabel™ DNA Labeling Kit (MBI Fermentas, Vilnius, Lithuania). BAC DNA was isolated using NucleoBond^® ^BAC 100 plasmid purification kit (Macherey-Nagel GmbH & Co. KG, Düren, Germany). Two overlapping BAC clones (68P2 and 109B10) containing *LHB/CGB *genome cluster were sheared by nebulization to approximately 5 kb long fragments and used for shotgun library construction with TOPO^® ^Shotgun Subcloning Kit (Invitrogen, Carlsbad, CA) according to manufacturers' instructions.

### DNA sequencing and data analysis

Plasmid DNA was purified with NucleoSpin^®^-Plasmid kit (Macherey-Nagel GmbH & Co. KG) and sequenced on ABI 3730 × l sequencer using BigDye^® ^Terminator v3.1 Cycle Sequencing Kit (Applied Biosystems, Foster City, CA). Plasmid ends were sequenced using M13F and M13R primers, additional primers for primer walking were designed with the web-based version of the Primer3 software [84]. Sequencing primers are available in Additional file 6. *LHB/CGB *genome cluster from two chimpanzee BAC clones were sequenced with an average redundancy of 7×, which was sufficient for assembly. Sequences were assembled using ContigExpress program from Vector NTI Suite 9 (Invitrogen) and the chimpanzee sequence was compared to human *LHB/CGB *genome cluster (GenBank: NG_000019). The full Chimpanzee *LHB/CGB *cluster sequence has been deposited to GenBank (accession number EU000308). Sequence alignments were performed and homologies determined by the web-based ClustalW [85] and Stretcher implemented in the EMBOSS package [86]. The aligned sequences of the major transcripts of the chimp and human *LHB/CGB *genes are given in Additional file 7. Substitution and indel divergences were calculated as the percentage of the number of substitutions and the number of nucleotides in indels divided with the total number of aligned nucleotides in the specific genomic region. Phylogenetic trees were constructed by MEGA3.1 [87] using Kimura's two parameter model to infer the neighbor-joining and the branch-and-bound algorithms to find maximum parsimony trees with 1000 replications for bootstrapping.

For coding regions maximum likelihood method [39] was used to estimate non-synonymous/synonymous rate ratio ω (= d_n_/d_s_) by CODEML implemented in PAML package version 4 [88,89]. Codon frequencies were estimated from the dataset using the F3 × 4 option, other settings were as default. The simplest model M0 or one-ratio model was used to estimate the ω (an average over all the sites). As for *CGB1 *alternative reading frames have been predicted and no functional protein has been characterized so far. We defined *CGB1 *mRNA sequence and subsequently the predicted reading frame as supported by the published experimental data [18,90]. In parallel, the number of non-synonymous substitutions per non-synonymous site (d_n_) and synonymous substitutions per synonymous site (d_s_) were estimated using an alternative method – the Li93 method [40,41]. The significance of the difference between d_n _and d_s _was examined by a two-tailed Z-test [42] using MEGA3.1. To address which segments of the genes are evolving more rapidly, we performed CRANN analysis [47,48] using sliding and overlapping windows and for the results in visual documentation of rate heterogeneities of d_n _and d_s_, Window size was set on 20 and shift size on 10 codons using the Li93 method.

Repetitive elements were detected by the REPEATMASKER program [91].

## Abbreviations

*ADH*: alcohol dehydrogenase; *CD33rSiglec*: CD33-related Siglecs; *CGB*: chorionic gonadotropin beta subunit gene; FAM90A: family with sequence similarity 90; GH/CSH: growth hormone/somatomammotropin; HCG: human chorionic gonadotropin; KGF: keratinocyte growth factor; LH: luteinizing hormone: LHB: luteinizing hormone beta subnit gene; MHC: major histocompatibility complex; NAIP: neuronal apoptosis inhibitory protein; NTF5: neurotrophin 5 gene; PRAME gene family: preferentially expressed antigen of melanoma; psNTF6: neurotrophin 6 pseudogene(s); ROCK1: Rho-dependent protein kinase; RUVBL2: RuvB-like 2, homologue of the bacterial RuvB gene; USP10: ubiquitin-specific protease.

## Authors' contributions

PH, JS, AP and ML designed the research; PH performed the research; JS, AP contributed to the analytic tools; PH and ML analyzed the data and wrote the paper. All authors read and approved the final manuscript.

## Supplementary Material

Additional file 1 Table for sequence parameters. Sequence parameters for the intergenic regions and for the whole *LHB/CGB *cluster in human and chimpanzee. Click here for file

Additional file 2 Distribution of species-specific indels. For simplification we defined all identified gaps in sequence alignments of human and chimpanzee *LHB/CGB *region orthologous segments (Figure 1B) as deletions in one of the species. The figure shows the number of species-specific gaps (Y-axes) relative to their length in base pairs (X-axis) and the contribution of each deletion class (1–5; 6–10; 11–15; 16–20; 20–25; >100 bp) to the total length of species-specific gapped sequence. Click here for file

Additional file 3 Table for nucleotide sequence divergence. Nucleotide sequence divergence in orthologous human and chimpanzee *LHB/CGB *genes. Divergence was estimated by using the human (GenBank: NG_000019) and chimpanzee (this study, Genbank: EU000308) reference sequences alone or by incorporating the re-sequencing data for one or both species (for human n = 95 [29]; for chimpanzee, n = 11, unpublished data of the authors) into the calculations. Click here for file

Additional file 4 Table of estimated d_n _and d_s _values by Li93 method. Application of Li93 method [40,41] for estimating non-synonymous (d_n_) and synonymous (d_s_) substitutions, and amino acid divergence in human and chimpanzee orthologous genes and for testing significance in the deviation of d_n_/d_s _ratio from expectation under neutrality. Click here for file

Additional file 5 Results of the CRANN analyses of human and chimpanzee orthologous genes (A) *LHB*, (B) *CGB5*, (C) *CGB8*, (D) *CGB7*, (E) *CGB1*. Results of moving window analysis carried out with CRANN [47,48]. X-axis shows the successive windows of 20 codon sites (window size: 20 codons, shift size: 10 codons). As the number of substitutions calculated in each moving window for human and chimpanzee orthologous genes was low, the d_n _and d_s _values were mostly zero and thus the d_n_/d_s _ratio was not shown. Click here for file

Additional file 6 Primer sequences. Primers for sequencing of subcloned BACs 68P2 and 109B10 originating from common chimpanzee (*Pan troglodytes*) BAC library RPCI-43 (BACPAC Resource Center at the Children's Hospital Oakland Research Institute; Oakland, CA). Click here for file

Additional file 7 Alignments of the chimp and human *LHB/CGB *transcripts. The aligned sequences of the major transcripts of the chimp and human *LHB/CGB *genes. The ATG of each gene has been indicated in red and underlined font. Translation STOP codons have been boxed. Click here for file
