# Supplementary material for: High divergence in primate-specific duplicated regions: Human and chimpanzee Chorionic Gonadotropin Beta genes
Source: BMC Evol Biol. 2008 Jul 7;8:195. doi: 10.1186/1471-2148-8-195 (PMC2478647; doi:10.1186/1471-2148-8-195)
Supplement: Additional file 1 — Table for sequence parameters. Sequence parameters for the intergenic regions and for the whole LHB/CGB cluster in human and chimpanzee. [file 1471-2148-8-195-S1.pdf]

Additional file 1.

Sequence parameters for the intergenic regions and for the total *LHB/CGB* cluster in human and chimpanzee.

		**Orthologous intergenic regions**					**Other regions**				
		**A**	**B**	**C**	**D**	**E**	***NTF5***	**B' human**	**B' chimp**	**C' human**	**Total *LHB/CGB* cluster**
**Region length (bp)**	**human/chimp**	5777/5890	6910/7511	2326/2315	5175/5168	5399/5387	2992/2986	7536	5357	2330	45165/39876
**GC %**		59/58	54/54	52/53	54/54	58/58	55/55	54	54	53	57/57
**CpG islands**	No. of islands	3/3	0/0	2/2	0/0	4/3	0/0	0	0	1	10/8
	Length (bp)	1102/1035	0/0	433/572	0/0	1201/830	0/0	0	0	232	2968/2437
	%	19/17.5	0/0	18.6/24.7	0/0	22.2/15.4	0/0	0	0	9.9	6.6/6.1
**Repeative sequences**											
SINEs: Alu&Mir	No. of elements	2/2	10/10	5/5	10/8	3/3	6/5	11	9	5	46/37
	Length (bp)	588/584	1934/2217	1289/1296	2327/2056	649/640	1322/1209	2404	1901	1286	10494/8696
	%	10.18/9.92	27.99/29.52	55.42/55.98	44.97/39.78	12.02/11.88	44.18/40.49	31.9	35.49	55.19	23.23/21.81
LINEs	No. of elements	0/0	2/1	0/0	2/1	0/0	0/0	2	1	0	6/3
	Length (bp)	0/0	282/134	0/0	179/134	0/0	0/0	268	134	0	729/402
	%	0/0	4.08/1.78	0/0	3.46/2.59	0/0	0/0	3.56	2.5	0	1.61/1.01
Satellites	No. of elements	1/1	0/0	0/0	0/0	1/1	0/0	0	0	0	2/2
	Length (bp)	93/93	0/0	0/0	0/0	130/89	0/0	0	0	0	223/182
	%	1.61/1.58	0/0	0/0	0/0	2.41/1.65	0/0	0	0	0	0.49/0.46
Simple repeats	No. of elements	4/5	2/3	2/1	1/1	1/1	0/1	2	2	2	14/13
	Length (bp)	135/178	66/104	56/23	36/33	186/186	0/33	64	68	65	608/592
	%	2.34/3.02	0.96/1.38	2.41/0.99	0.7/0.64	3.45/3.45	0/1.11	0.85	1.27	2.79	1.35/1.48
Low complexity	No. of elements	0/1	1/1	1/1	0/0	0/0	2/1	0	1	0	2/4
	Length (bp)	0/38	23/21	77/77	0/0	0/0	129/51	0	21	0	100/157
	%	0/0.65	0.33/0.28	3.31/3.33	0/0	0/0	4.31/1.71	0	0.39	0	0.22/0.39
Total	Length (bp)	816/893	2305/2476	1422/1396	2542/2223	965/915	1451/1293	2736	2124	1351	12154/10029
	%	14.13/15.17	33.36/32.96	61.14/60.3	49.13/43.01	17.88/16.98	48.49/43.31	36.31	39.65	57.98	26.9/25.15

**Indels**						
	**No. of indels**	14	23	10	12	2
	**Bp in indels**	212	713	23	47	14
	**Minimum length**	1	1	1	1	1
	**Maximum length**	128	637	7	16	13
	**Average length**	15.1	31	2.3	3.9	7
**Divergence**						
	**Indel divergence %**	3.5	9.4	1	0.9	0.3
	**Substit. divergence %**	1.3	3.6	2.9	2.9	1.3
	**Total divergence %**	4.8	13	3.9	3.8	1.6

Indel divergence % - nucleotides in indels divided by the total number of aligned nucleotides x100.
Total divergence - the sum of indel divergence and substitution divergence
CpG islands - sequence stretches of >200 bp, GC content of >50% and the observed to expected CpG ratio of >0.6
