# Supplementary figures and images for: High divergence in primate-specific duplicated regions: Human and chimpanzee Chorionic Gonadotropin Beta genes
Source: BMC Evol Biol. 2008 Jul 7;8:195. doi: 10.1186/1471-2148-8-195 (PMC2478647; doi:10.1186/1471-2148-8-195)

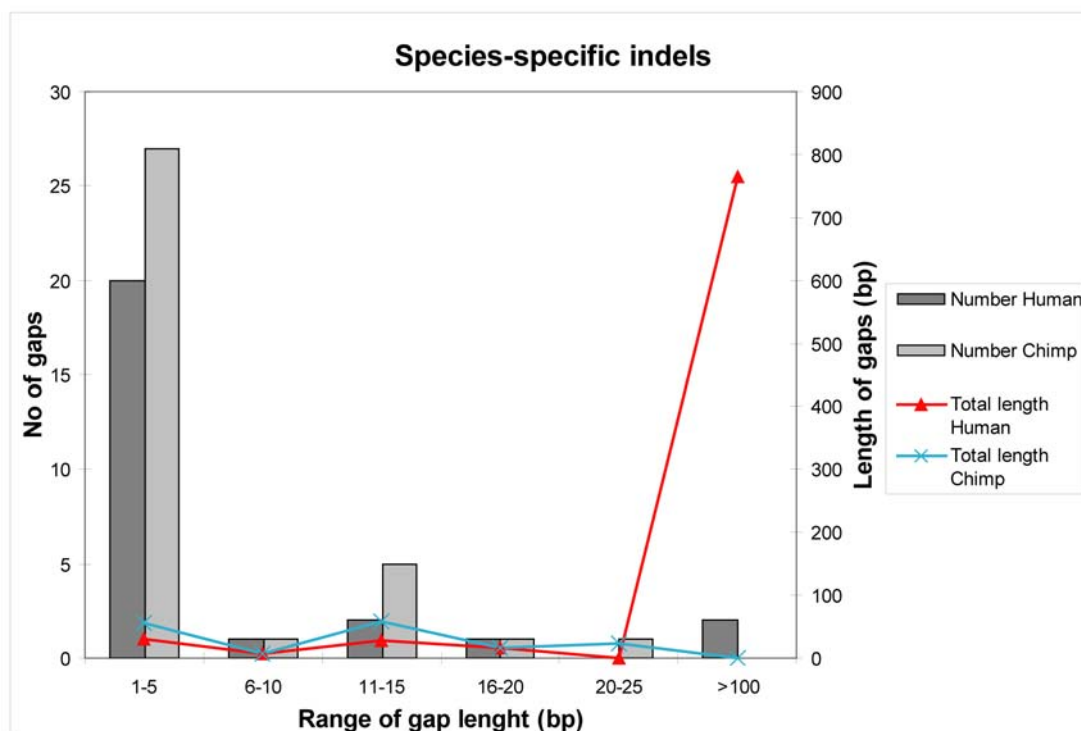

Supplement: Additional file 2 — Distribution of species-specific indels. For simplification we defined all identified gaps in sequence alignments of human and chimpanzee LHB/CGB region orthologous segments (Figure 1B) as deletions in one of the species. The figure shows the number of species-specific gaps (Y-axes) relative to their length in base pairs (X-axis) and the contribution of each deletion class (1–5; 6–10; 11–15; 16–20; 20–25; >100 bp) to the total length of species-specific gapped sequence. [file 1471-2148-8-195-S2.pdf]

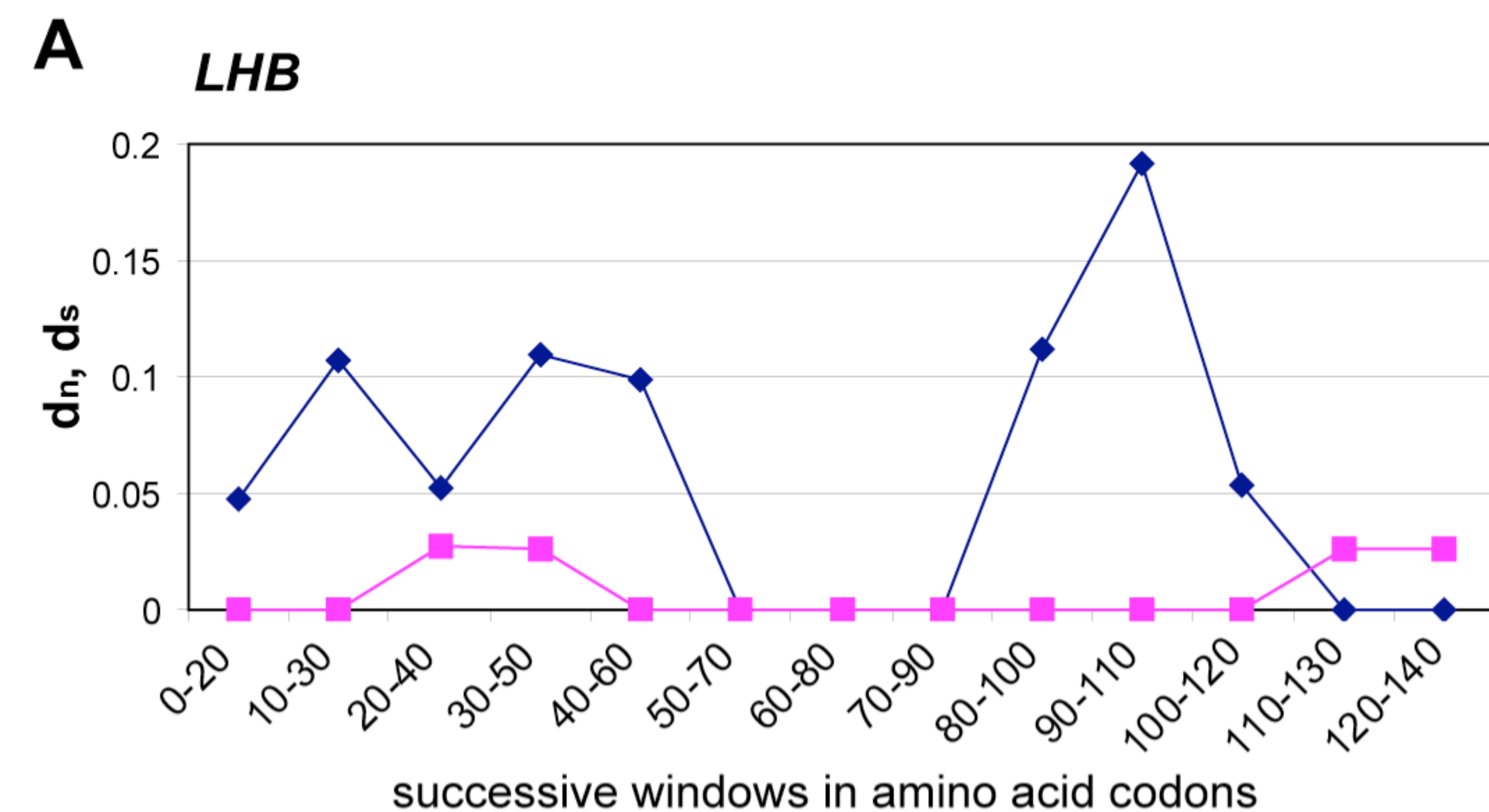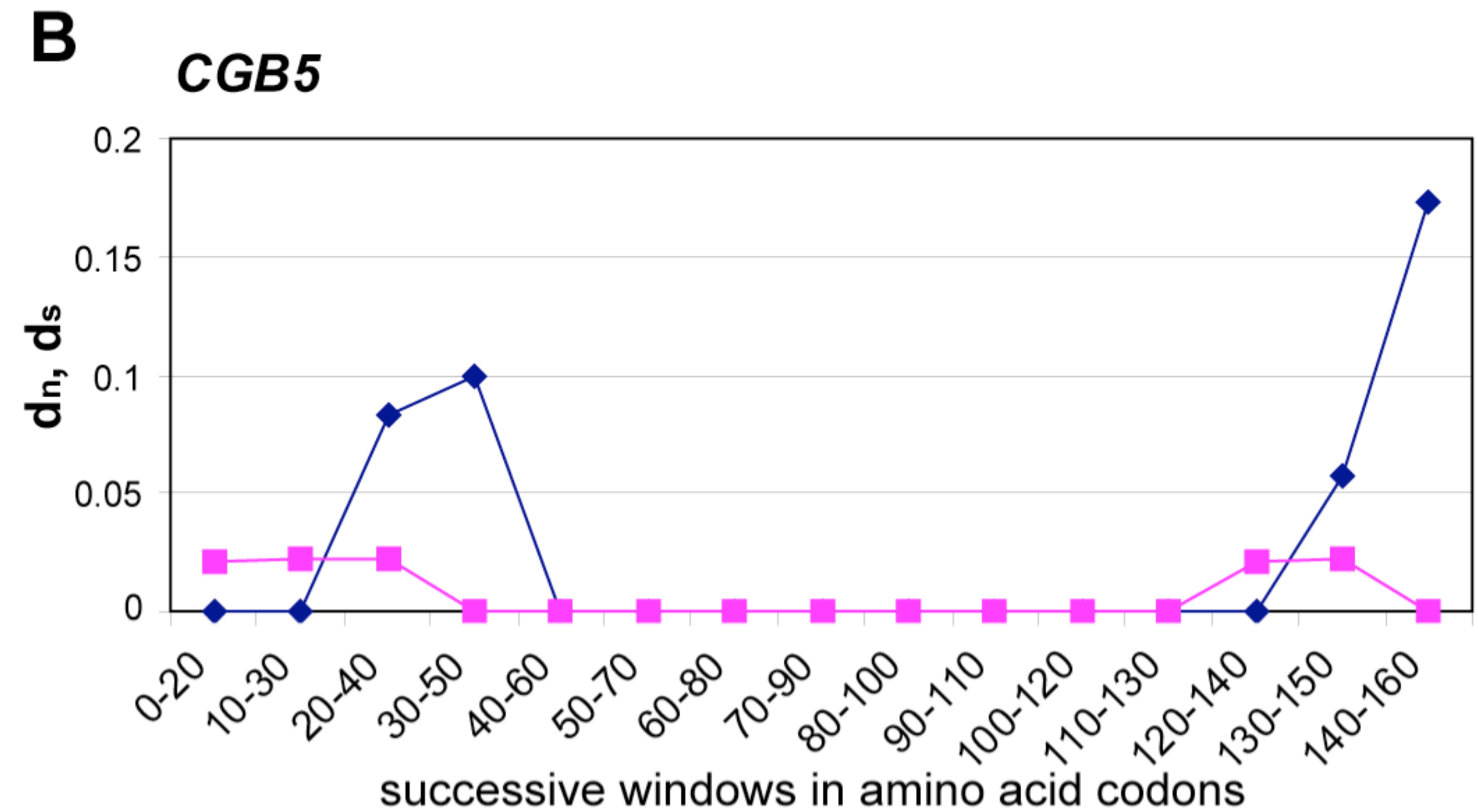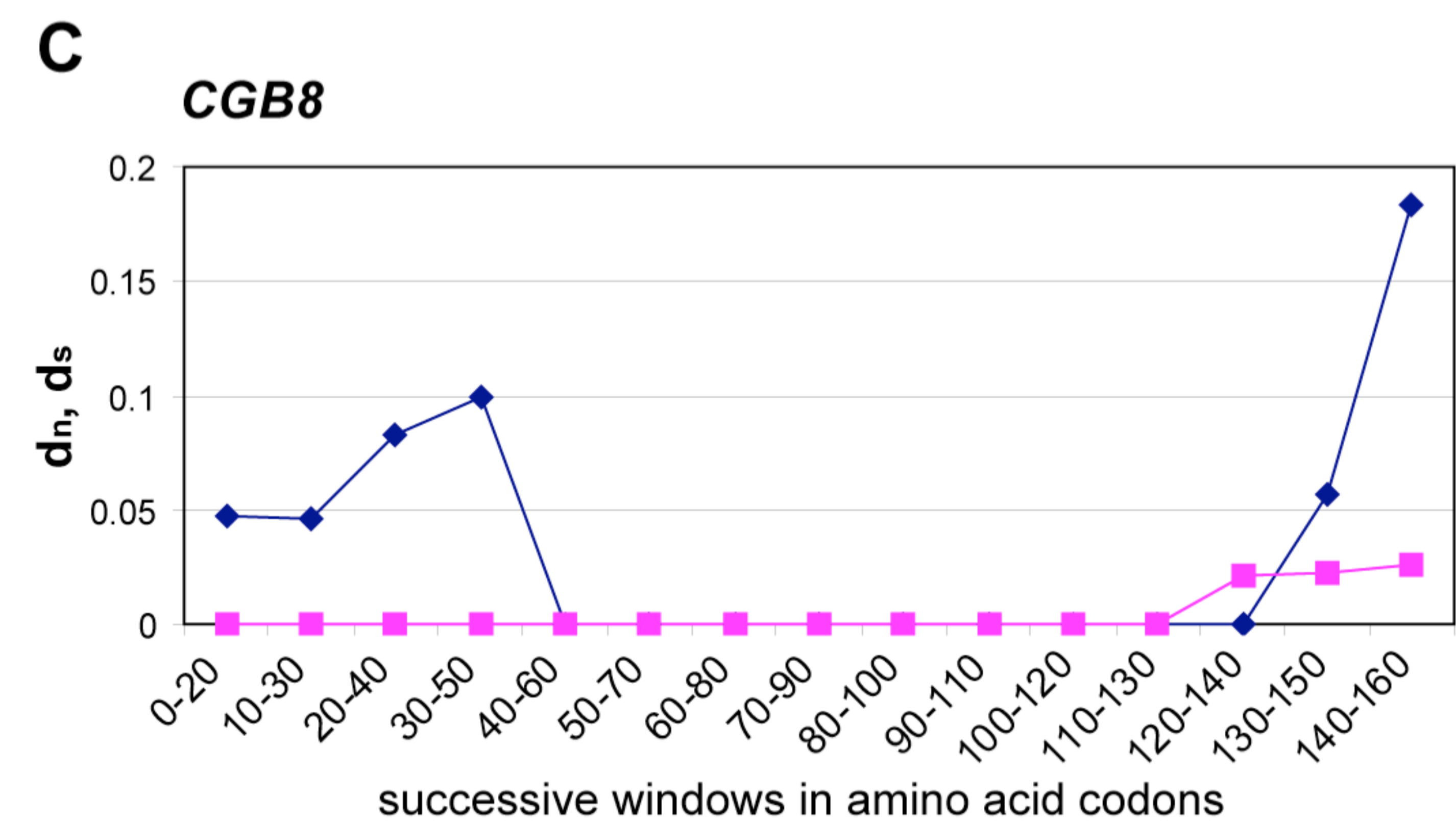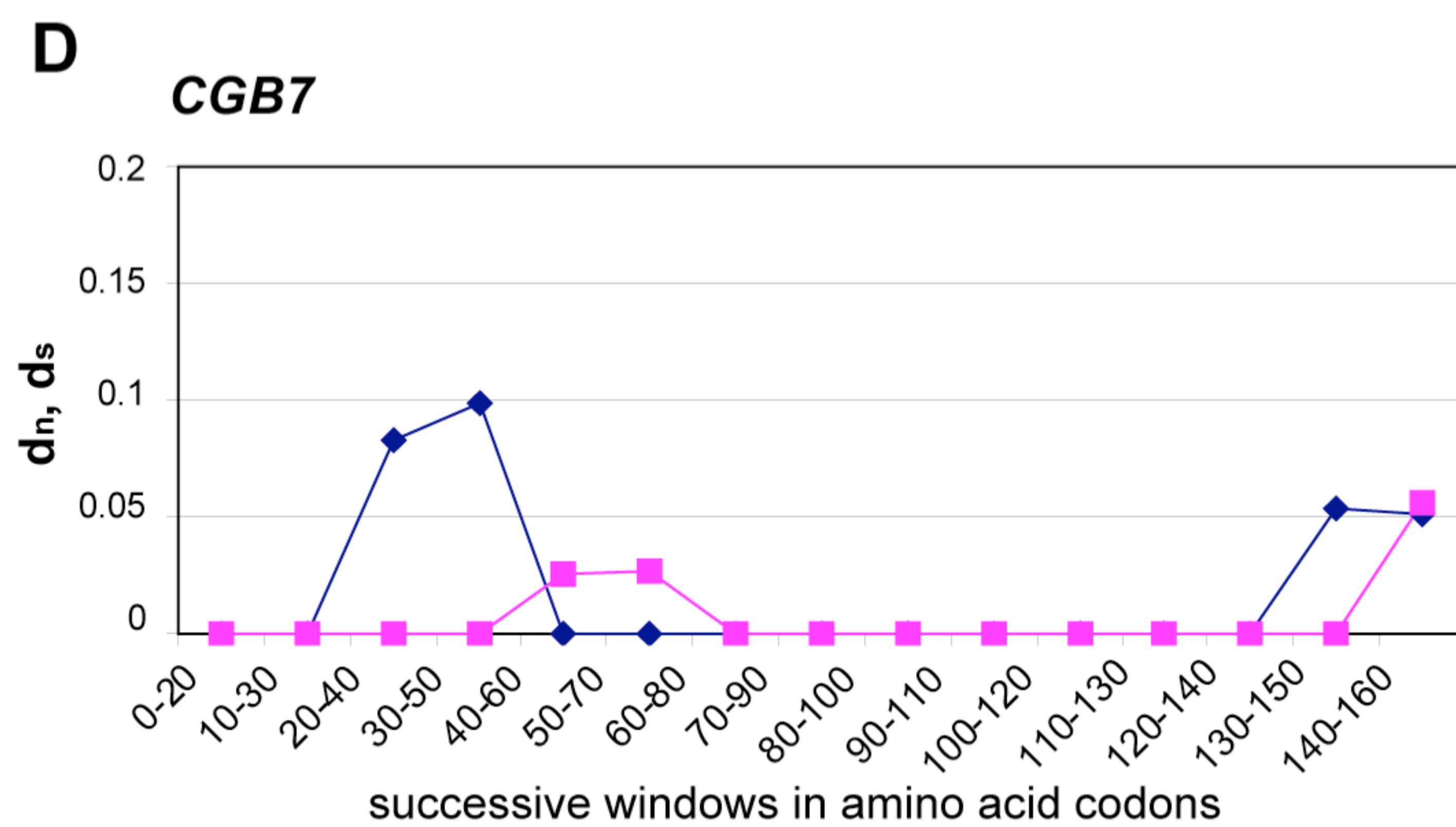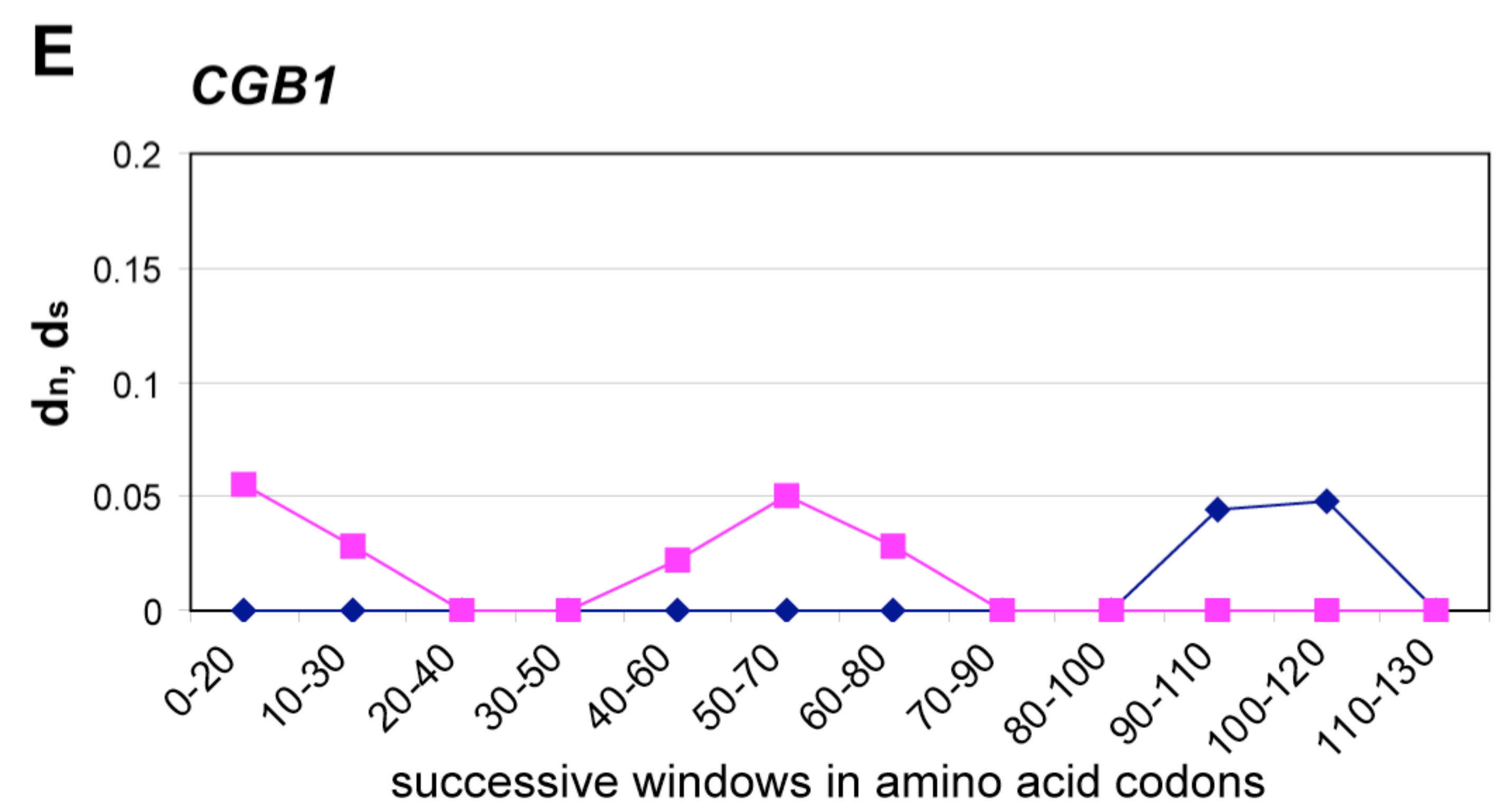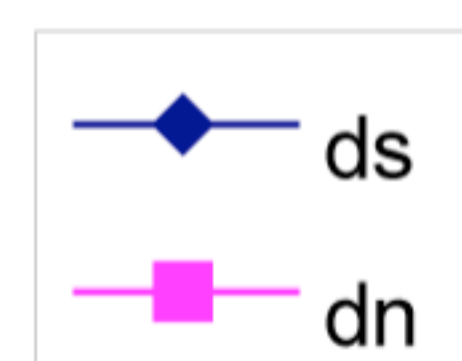

Supplement: Additional file 5 — Results of the CRANN analyses of human and chimpanzee orthologous genes (A) LHB, (B) CGB5, (C) CGB8, (D) CGB7, (E) CGB1. Results of moving window analysis carried out with CRANN [47,48]. X-axis shows the successive windows of 20 codon sites (window size: 20 codons, shift size: 10 codons). As the number of substitutions calculated in each moving window for human and chimpanzee orthologous genes was low, the dn and ds values were mostly zero and thus the dn/ds ratio was not shown. [file 1471-2148-8-195-S5.pdf]
