# Supplementary material for: High divergence in primate-specific duplicated regions: Human and chimpanzee Chorionic Gonadotropin Beta genes
Source: BMC Evol Biol. 2008 Jul 7;8:195. doi: 10.1186/1471-2148-8-195 (PMC2478647; doi:10.1186/1471-2148-8-195)
Supplement: Additional file 3 — Table for nucleotide sequence divergence. Nucleotide sequence divergence in orthologous human and chimpanzee LHB/CGB genes. Divergence was estimated by using the human (GenBank: NG_000019) and chimpanzee (this study, Genbank: EU000308) reference sequences alone or by incorporating the re-sequencing data for one or both species (for human n = 95 [29]; for chimpanzee, n = 11, unpublished data of the authors) into the calculations. [file 1471-2148-8-195-S3.pdf]

Additional file 3.
Nucleotide sequence divergence in orthologous *LHB/CGB* genes between human and chimpanzee.

		Length (bp)	Chimpanzee reference vs Human reference[a]		Chimpanzee reference vs human population data[b]		Chimpanzee population data vs Human reference[c]		Chimpanzee population data vs human population data[d]	
**Gene**	**Region**	**human/chimp**	**Fixed diff.[e]**	**Div % [f]**	**Fixed diff.[e]**	**Div % [f]**	**Fixed diff.[e]**	**Div %[f]**	**Fixed diff.[e]**	**Div %[f]**
LHB	Promoter	356	7	2	n.a.	n.a.	n.a.	n.a.	n.a.	n.a.
	5'UTR	9	0	0	0	0	0	0	0	0
	gene	1111	20	1.8	16	1.44	18	1.62	14	1.26
	mRNA	523	8	1.53	6	1.15	7	1.34	5	0.95
	exons	426	8	1.88	6	1.41	7	1.64	5	1.17
	introns	588	12	2.04	10	1.7	11	1.87	9	1.53
	3'UTR	88	0	0	0	0	0	0	0	0
CGB1	Promoter	739/736	38 (+3bp del)	5.1 (5.5)	n.a.	n.a.	n.a.	n.a.	n.a.	n.a.
	5'UTR	174/176	4 (+2bp ins)	2.3 (3.4)	4 (+2 ins)	2.3 (3.4)	n.a.	n.a.	n.a.	n.a.
	gene	1366/1368	33 (+ 2bp ins)	2.41 (2.56)	26 (+2 ins)	1.9 (2.05)	n.a.	n.a.	n.a.	n.a.
	mRNA	732/734	18 (+2bp ins)	2.45 (2.72)	14 (+2 ins)	1.91 (2.18)	n.a.	n.a.	n.a.	n.a.
	exons	399	5	1.25	4	1	n.a.	n.a.	n.a.	n.a.
	introns	634	15	2.36	12	1.89	n.a.	n.a.	n.a.	n.a.
	3'UTR	159	9	5.66	6	3.77	n.a.	n.a.	n.a.	n.a.
CGB5	Promoter	311/310	14 (+1bp del)	4.5 (4.8)	n.a.	n.a.	n.a.	n.a.	n.a.	n.a.
	5'UTR	365	14	3.83	12	3.29	12	3.29	10	2.74
	gene	1467	38	2.59	29	1.98	36	2.45	27	1.84
	mRNA	880	21	2.38	18	2.05	18	2.05	15	1.7
	exons	498	7	1.4	6	1.2	6	1.2	5	1
	introns	587	17	2.89	11	1.87	18	3.06	12	2.04
	3'UTR	17	0	0	0	0	0	0	0	0
CGB8	Promoter	310	11	3.5	n.a.	n.a.	n.a.	n.a.	n.a.	n.a.
	5'UTR	365	12	3.28	11	3.01	12	3.28	11	3.01

	gene	1467	38	2.59	34	2.32	32	2.18	28	1.9
	mRNA	880	19	2.16	18	2.04	16	1.81	15	1.7
	exons	498	7	1.4	7	1.4	4	0.8	4	0.8
	introns	587	19	3.24	16	2.72	16	2.72	13	2.21
	3'UTR	17	0	0	0	0	0	0	0	0
CGB7	Promoter	311	3	1	n.a.	n.a.	n.a.	n.a.	n.a.	n.a.
	5'UTR	365	12	3.29	12	3.29	12	3.29	11	3.01
	gene	1467	32	2.18	25	1.7	31	2.11	23	1.57
	mRNA	880	17	1.93	17	1.93	16	1.81	15	1.7
	exons	498	5	1	5	1	4	0.8	4	0.8
	introns	587	15	2.55	8	1.36	15	2.55	8	1.36
	3'UTR	17	0	0	0	0	0	0	0	0

[a] Divergence between chimpanzee EU000308 and human reference sequence NG_000019

[b] Divergence between chimpanzee EU000308 and human population data from a re-sequencing study (3 populations, n = 95) (Hallast et al. 2005), for *CGB8* gene a re-sequencing data (1 population, n = 194) from (Rull et al. 2008)

[c] Divergence between chimpanzee population data (n=11, unpublished data) and human reference sequence NG_000019

[d] Divergence between chimpanzee population data (n=11, unpublished data) and human population data from a re-sequencing study (3 populations, n = 95) (Hallast et al. 2005), for *CGB8* gene a re-sequencing data (1 population, n = 194) from (Rull et al. 2008)

[e] The number of fixed differences between species. In brackets are shown indels found in chimp.

[f] Nucleotide divergence (%). In brackets are shown nucleotide divergence including indels.

n.a. – not applicable i.e. sequence data is not available
