# Supplementary material for: High divergence in primate-specific duplicated regions: Human and chimpanzee Chorionic Gonadotropin Beta genes
Source: BMC Evol Biol. 2008 Jul 7;8:195. doi: 10.1186/1471-2148-8-195 (PMC2478647; doi:10.1186/1471-2148-8-195)
Supplement: Additional file 4 — Table of estimated dn and ds values by Li93 method. Application of Li93 method [40,41] for estimating non-synonymous (dn) and synonymous (ds) substitutions, and amino acid divergence in human and chimpanzee orthologous genes and for testing significance in the deviation of dn/ds ratio from expectation under neutrality. [file 1471-2148-8-195-S4.pdf]

Additional file 4.

d_n and d_s values and amino acid divergence in orthologous genes between human and chimpanzee using Li93 method (Li 1993; Pamilo and Bianchi, 1993)

Gene	d_n	d_s	d_n/d_s	p-value[a]	AA %[b]
LHB	0.007	0.048	0.146	0.049*	1.42
CGB1	0.015	0.007	2.143	0.354	3.03
CGB5	0.008	0.031	0.258	0.16	1.82
CGB8	0.006	0.037	0.162	0.078	1.21
CGB7	0.01	0.016	0.625	0.625	1.82

[a] *p*-values for $d_n = d_s$ with Z-test, $*p<0.05$

[b] Divergence at amino acid level (%)
