# Supplementary material for: High divergence in primate-specific duplicated regions: Human and chimpanzee Chorionic Gonadotropin Beta genes
Source: BMC Evol Biol. 2008 Jul 7;8:195. doi: 10.1186/1471-2148-8-195 (PMC2478647; doi:10.1186/1471-2148-8-195)
Supplement: Additional file 6 — Primer sequences. Primers for sequencing of subcloned BACs 68P2 and 109B10 originating from common chimpanzee (Pan troglodytes) BAC library RPCI-43 (BACPAC Resource Center at the Children's Hospital Oakland Research Institute; Oakland, CA). [file 1471-2148-8-195-S6.pdf]

Additional file 6.
List of sequencing primers.

Sequencing primer	Primer sequence 5'-3'
CGB_1F	CAATGTAGAGTAAAGTTGCGATGTC
CGB_3R	AGTTAAATCACCTGAAGCACAC
CGB1_1F	GTTGGTCTGGAACCCCTCA
CGB1_2R	ATGGAGCCAATCACAAGAGG
CGB2_1F	AGGAGAGGCTCACCCCTGAC
CGB2_2R	CCCGGATAACTTTTCGTATTTTTA
CGB5_3F	CAGGAAAGCCTCAAGTAGAGGAG
CGB5_2R	CGCTCGACGATGTTTTCTATTTT
CGB7_1F	CCCCATCTTCACTCTCTTTTCTT
CGB7_3R	GGCTCGCACTCATTAAGTCTATC
LHB_1F	ATGACATCAAGCGGGTCTAC
LHB_4R	GGATTAGTGTCCAGGTTACCC
CGBantisense_1seqR	GTCAACACCACCATCTGTGC
CGBantisense_2seqF	GTCCAGGAAGCCCTCTGTT
CGBantisense_2seqR	CACCTTCCACCTCCTTCCAG
CGBantisense_3seqF	TCCTATTCAGGACCCACCAC
CGBsense_1seqR	TGGTACACCACCCACAAAGA
CGBsense_2seqF	CTCTTTCTGGAGGAGCGTGA
CGBsense_2seqR	ACATCGCGGTAGTTGCACA
CGBsense_3seqF	CCCCTGAGTCTGAGACCTGT
CGB2_5R	ACCGGCTAGGGGAAGAAAAGAAC
cgb5Rnest	AGCCCTCCTTCTCCACAGC
CGB1_3F	CAGGGATGAGCCTCTTGTGATT
cgb7Rmulti	GGCCTTTGAGGAAGAGGAG
gor-L_1F1Rvahel	CCACCAGAGTTCTGTACTGTGAC
CGB2prom1F	CGAGTAGTGGGACATCCCACTTGCT
simp-c1-2Fasemel	AGAGGGAGACCACCCTTCCT
c1_promRlisa2seq	GTCATTTCTTCCTATCAAGG
II_frag_rv	ACACCCCGATCCTCCCACAATAAAG
LHB_huhF3	CGGGGGCAAGACACGCA
LH_9RpooleF	GCTTCTGCCCAGTGAGAGAG
VI_frag_rv	CGCACTCTGGAGGTGTCTTTCTGT
VII_frag_fw	TCTACTTGAGGCTTTCCTGCACCACA
CGB2_6R	TCCTGGAACATCTCCATCTTTGG
CGB-RseqRNA	TGGAACATCTCCATCCTTGG
CGB/CGBII_rv	GAGGACGCCTTGATAGGAAGAAATG
CGB2_4R	TTTTAGATCCCCACCCTCAGGAA
gor-c2_2Fasemel	GAAGGGTCTCTGGGTCTTTGT
CGB1_2F	TCTACAGGAAGGATGGCAGAGTG
simp-c1-3Rasemel	GGAAATGTGGATCTACCCTACCT
simp-c1_1F1Rvahel	ATCACAGGTCAAGGGGTGGT
simp-L-1F1Rvahel	AGCTGGACCCACCCTATGTAT
gor-c2-2Rasemel	GTCTGGAAGCCGTGTGAGA
CGB2prom2F	CCTATCAAGGCGTCCTCCCTTTA
CGB1_3R	TAAAGGCTTCTCAATCCCTCTGG
CGB1_4R	CTTTGATCTTACGCAGGGTGATG
CGB1_5R	CATTCTGTTTACCACAGGTGACGA
CGB1_6R	ATCACAAGAGGCTCATCCCTGAC
CGB1_4F	ATTGGCTGCTCTCTCTCAGATGC
C2_promFlisa1seq	AGTAATTTAAAAACACTTAGG
C2_promFlisa2seq	GTCATTTCTTCCTATCAAGG
C2_promRlisa1seq	ACAAAACATAAAGGGAGGAC
C2_promFlisa3seq	ACCAGGTTGGCCTCGAACT

C1_promRlisa1seq	GCCCAGGTTGACCTCGAA
c1_promFlisa1seq	CAACAAAACATAAAGGGAGGAC
C1_5Rseq	TGTTTACCACAGGTGACGAC
CGB5/7_nest4_sekv2_F	CATAGGACTGCTTGGCCTTG
CGB5/7_nest4_sekv2_R	TTCCAACTTCTGACCCCACT
F_CGB5-7_nest2_1	GCAGGGTACCCAAGAGTCAA
CGB5/7_nest6_sekv1_R	AACCACAGGCTTCCAGAATG
CGB5/7_nest1_sekv3_F	CCTCGGCCTCCCAAGTAG
CGB5/7_nest2_sek2_R2	GCCTCAGGTGGTGTGCAA
CGB5/7_nest5_sekv2_F	CTGCAATCCCAGCACTATGG
CGB5/7_nest3_sekv3_F	GCCCACTCTACCCTCAAGC
CGB5/7_nest4_sekv1_R	CTTCGAGGCCGATAACTCTG
CGB5/7_nest4_sekv3_F	GGGAGCGGGAGCATCTCT
CGB5/7_nest6_sekv2_R	CTCCTTCATCCACTGCCTCT
CGB5/7_nest6_sekv2_F	GGAGACCCTGATTCACCTCA
CGB5/7_nest3_sekv1_R	GCTGGACCAGTGAGAGGAGA
R_CGB5-7_nest4_1	CTGGAACTCCTCACCTCAGC
F_CGB5-7_nest2_1	GCAGGGTACCCAAGAGTCAA
F_CGB5-7_nest5_2	GATCGTGTCACTGCACTTCG
CGB5/7_nest2_sekv1_R	TCATGAGGTCAGGAGATCGAG
CGB5/7_nest5_sekv1_R	AGCTGGGACTACAGGTGTGC
CGB5/7_nest5_sekv2_R	TAAGGCAGGAGAATGGCTTG
R_CGB5-7_nest3_1	TCCTCAGATCAACTCTCATGGAT
CGB5/7_nest2_sekv2_F	ACTGCATTCTCCTGCAAGC
CGB5/7_nest1_sekv1_F	GTGGGTGGATCAGCTGAGGT
CGB5/7_nest1_sek2_F2	AGTTCCAGCTACTCAGGAAGC
F_CGB5-7_nest4_1	AGTTTCTCTCCACCCCCATC
CGB5/7_nest3_sekv2_F	GGAGTGAGCTCGACACTAACC
CGB5/7_nest1_sekv2_R	GATCACAAAGTCAGGAGATCAAG
CGB5/7_nest6_sekv3_F	GCAGGGTACCCAAGAGTCAA
R_CGB5-7_nest1_1	TCCTTCATCCACTGCCTCTC
CGB5/7_nest1_sekv1_R	GAGACGGAGTCTCACCCTGT
CGB5/7_nest1_sekv3_F	CCTCGGCCTCCCAAGTAG
8kb-F3	CACGCCTGTAATTGTCGGAGGCTGT
8kb-R3	GAAAAGAGAGTGAAGATGGGGGACGAC
13-T3-1	CTGGCTTGAGGGTAGAGTGG
61-T7-1	CTTTGCTCAAAGCTGGACCT
61-T3-1	GTGTTTGCACACTCCTCAGC
252-T3-1	CAGGCGCTTGTAATTCCAGT
252-T7-1	TGGTCCACATTGGTAGGACA
256-T3-1	GCTGGGGTTCAAATGAGAAA
5-T3-1	CCCCTAGACAGGACCACTCA
77-T7-1	GGCACTCCCCAAACTTTACC
58-T3-1	TGAAGGGTATGGGTGGGATA
67-T7-1	CTGCTTCCTCCCTCTACCAG
102-T3-1	GTGGCGCACACCTGTAATC
92-T7-1	GGGTTCACACCACTCTCCTG
252-T3-2	CTTGGCTCACTGCATCTCTG
502-T7-1	ACTCCTGAGCCAGAGACAGG
502-T7-2	GGGTGGGGAGGAGACTAGAA
502-T7-3	CAGCAGATGGGAGACAACCT
61-T7-2	TCCTGTAATCCCAGCACTTTG
102-T3-2	CCCTTGAGTAGGGTCAGCAA
445-T7-1	TTACAGTGAGCCGGGATTGT
84-T7-1	CTGGTCACACAGCCAACAAA
84-T3-1	CCTGTATTTTACCATCTGTTGC
466-T3-2	CAAATGGCAGAGATGGGACT
466-T3-3	TCCAAGATGCAAGACTGTCG

466-T3-4	CGCATGTCTGTAATCCCAGTT
466-T7-2	GCGATGGGTAACATGTGAAG
VIII-fr-F1	CCTGGAGGAGCAGAGACAGG
VIII-fr-R	GATTGGGAGCTTGGACTCCTCT
VIII-fr-F2	GGTTTCCTGAGCCAGAGACAT
5-T3-2	ACTCAATAGCAACAGATGGTAAAA
5-T3-3	CTGAGGCAGGAGAGTGGTGT
CGB2_3R	CCACCCTCTTTTCTTTTTCTTTCT
445-T7-3	CTGCAGTCCCAGCTACTCG
438-t3-1	CGGGAGAGCTCCTAGTCAAG
466-T7-1	TCGGTTGCAGAGATGAAAGA
445-T3-1	CTGAGGCAGGTGAATTGCTT
445-T7-2	ATGCCTGTAATCCCAGCACT
25-T7-1	ACACCAAAGGTCACCAATGA
84-T3-2	CCTGGGCAACAAGTGAGACT
84-T3-3	GCAGTCCAGTCTCAACAACAGA
502-T7-4	GTGGACTCAGCCTCGACTTC
84-T3-4	ATGTTCAGCCAGGCGCAGT
CGB5_simp1R	ACTGCGCCTGGCTGAACAT
CGB8-8kb_simpF3	CACGCCTGTAATTGTCGGAGGCTGT
CGB8-8kb_simpR3	GAAAAGAGAGTGAAGATGGGGGATGAC
N4S3F_simp1	CAAGCTACTTGGGAGGCTGA
N6S2F_simp1	AACAACCCCTTCCCAGAGTT
466-T3-1	ATTGCTTGAGGTTGGGAGTT
466-T7-3	CTTGAGTGGGGCATGAGTTT
466-T7-4	CTGGCCCTACTCAACAGCTC
466-T3-5	TTACAGGTGTGAGCCACCAT
gor-LHB_2F	AGGACGCCTTCCTCTTCAAT
466-T7-5	TGAACCCCATCTCCCAGATA
466-T3-6	CCCTGAGTGGGACCTTCTCT
