# Supplementary material for: High divergence in primate-specific duplicated regions: Human and chimpanzee Chorionic Gonadotropin Beta genes
Source: BMC Evol Biol. 2008 Jul 7;8:195. doi: 10.1186/1471-2148-8-195 (PMC2478647; doi:10.1186/1471-2148-8-195)
Supplement: Additional file 7 — Alignments of the chimp and human LHB/CGB transcripts. The aligned sequences of the major transcripts of the chimp and human LHB/CGB genes. The ATG of each gene has been indicated in red and underlined font. Translation STOP codons have been boxed. [file 1471-2148-8-195-S7.pdf]

```
LHB_human      1 ------------------------------------------------------------
LHB_chimp      1 ------------------------------------------------------------
CGB_human      1 TCCAGCACCTTTCTCGGGTCACGGCCTCCTCCTGGCTCCCAGGACCCCACCATAGGCAGA
CGB5_human     1 TCCAGCACTTTGCTCGGGTCACGGCCTCCTCCTGGCTCCCAGGACCCCACCATAGGCAGA
CGB5_chimp     1 TCCAGCACTTTGCTCGGGTCACGGCCTCCTCCTGGCTTCCAAGACCCCACCATAGGCAGA
CGB8_human     1 TCCAGCACCTTTCTCGGGTCACGGCCTCCTCCTGGCTCCCAGGACCCCACCATAGGCAGA
CGB8_chimp     1 TCCAGCACTTTGCTCGGGTCACGGCCTCCTCCTGGCTTCCAAGACCCCACCATAGGCAGA
CGB7_human     1 TCCAGCACCTTTCTCGGGTCACGGCCTCCTCCTGGTTCCCAAGACCCCACCATAGGCAGA
CGB7_chimp     1 TCTAGCACCTTTCTCGGGTCACGGCCTCCTCCTGGTTCCCAAGACCCCACCATAGGCAGA
CGB2_human     1 ------------------------------------------------------------
CGB1_human     1 ------------------------------------------------------------
CGB1_chimp     1 ------------------------------------------------------------
CGB1B_chimp    1 ------------------------------------------------------------

LHB_human      1 ------------------------------------------------------------
LHB_chimp      1 ------------------------------------------------------------
CGB_human     61 GGCAGGCCTTCCTACACCCTACTCCCTGTGCCTCCAGCCTCGACTAGTCCCTAGCACTCG
CGB5_human    61 GGCAGGCCTTCCTACACCCTACTCCCTGTGCCTCCAGGCTCGACTAGTCCCTAGCACTCG
CGB5_chimp    61 GGCAGGCCTTCCTACACCCTACTCCCTGTGCCTCCAGCCTTGACTAGTCCCTAGCACTCG
CGB8_human    61 GGCAGGCCTTCCTACACCCTACTCCCTGTGCCTCCAGGCTCGACTAGTCCCTAGCACTCG
CGB8_chimp    61 GGCAGGCCTTCCTACACCCTACTCCCTGTGCCTCCAGCCTCGACTAGTCCCTAGCACTCG
CGB7_human    61 GGCAGGCCTTCCTACACCCTACTCTCTGTGCCTCCAGCCTCGACTAGTCCCTAGCACTCG
CGB7_chimp    61 GGCAGGCCTTCCTACACCCTACTCCCTGTGCCTCCAGCCTCGACTAGTCCCTAGCACTCG
CGB2_human     1 ------------------------------------------------------------
CGB1_human     1 ------------------------------------------------------------
CGB1_chimp     1 ------------------------------------------------------------
CGB1B_chimp    1 ------------------------------------------------------------

LHB_human      1 ------------------------------------------------------------
LHB_chimp      1 ------------------------------------------------------------
CGB_human    121 ACGACTGAGTCTCTGAGGTCACTTCACCGTGGTCTCCGCCTCACCCTTGGCGCTGGACCA
CGB5_human   121 ACGACTGAGTCTCTGAGATCACTTCACCGTGGTCTCCGCCTCACCCTTGGCGCTGGACCA
CGB5_chimp   121 ACGACTGAGTCTCTGAGGTCACTTCACCGTGGTCTCCGCCTAACCCTTGGCGCTGGACCA
CGB8_human   121 ACGACTGAGTCTCTGAGGTCACTTCACCGTGGTCTCCGCCTCACCCTTGGCGCTGGACCA
CGB8_chimp   121 ACGACTGAGTCTCTGAGGTCACTTCACCTTGGTCTCCGCCTCACCCTTGGCGCTGGACCA
CGB7_human   121 ACGACTGAGTCTCAGAGGTCACTTCACCGTGGTCTCCGCCTCATCCTTGGCGCTAGACCA
CGB7_chimp   121 ACGACTGAGTCTCTGAGGTCACTTCACCGTGGTCTCTGCCTCATCCTTGGCGCTAGACCA
CGB2_human     1 ----------CCCCAGGGCCAGT-----GAGGGCCCTGCGT--TCCGTGGCGC----CCC
CGB1_human     1 ----------CCCCAGGGCCAGT-----GAGGGCCCTGCGT--TCCGTGGCGC----CCC
CGB1_chimp     1 ----------CCCCAGGGCCAGT-----GAGGGCCCTGTGT--TCCGTGGCGC----CCC
CGB1B_chimp    1 ----------CCCCAGGGCCAGT-----GAGGGCCCTGTGT--TCCGTGGCGC----CCC

LHB_human      1 ------------------------------------------------------------
LHB_chimp      1 ------------------------------------------------------------
CGB_human    181 GTGAGAGGAGAGGGCTGGGGCGCTCCGCTGAGCCACTCCTGCGCCCCCCTGGCCTTGTCT
CGB5_human   181 GTGAGAGGAGAGGGCTGGGGCGCTCCGCTGAGCCACTCCTGCGCCCCCCTGGCCTTGTCT
CGB5_chimp   181 CCGAGAGGAGAGGGCTGGGGCGCTTCGCTGAGCCACTCCTGCAACCCCCTGGCCTTGTCT
CGB8_human   181 GTGAGAGGAGAGGGCTGGGGCGCTCCGCTGAGCCACTCCTGCGCCCCCCTGGCCTTGTCT
CGB8_chimp   181 CTGAGAGGAGAGGGCTGGGGCGCTCCGCTGAGCCACTCCTGCAACCCCCTGGCCTTGTCT
CGB7_human   181 CTGAGGGGAGAGGACTGGGGTGCTCCGCTGAGCCACTCCTGTGCCTCCCTGGCCTTGTCT
CGB7_chimp   181 CCGAGGGGAGAGGGCTGGGGCGCTCCGCTGAGCCACTCCTGTGCCTCCCTGGCCTTGTCT
CGB2_human    40 CTGGAGGGAGGAAGGGGAACTGTATCTGAGAGAGA--GCAGCCAATTGGGTCCGCTGACT
CGB1_human    40 CTGGAGGGAGGAAGGGGAACTGCATCTGAGAGAGA--GCAGCCAATTGGGTCCGCTGACT
CGB1_chimp    40 CTGGAGGGAGGAAGGGGAACTGTATCTGAGAGAGAGAGCAGCCAATTGGGTCCGCTGACT
CGB1B_chimp   40 CTGGAGGGAGGAAGGGGAACTGTATCTGAGAGAGAGAGCAGCCAATTGGGTCCGCTGACT

LHB_human      1 ------------------------------------------------------------
LHB_chimp      1 ------------------------------------------------------------
CGB_human    241 ACCTCTTGCCCCCCGAGGGGTTAGTGTCGAGCTCACCCCAGCATCCTATCACCTCCTGGT
CGB5_human   241 ACCTCTTGCCCCCCGAAGGGTTAGTGTCGAGCTCACCCCAGCATCCTACAACCTCCTGGT
CGB5_chimp   241 ACCTCTTGCCCCCCGAAGGGTTAGTGTCGAGCTCACCCCAGCATCCTACAACCTCCTGGT
CGB8_human   241 ACCTCTTGCCCCCCGAAGGGTTAGTGTCGAGCTCACTCCAGCATCCTACAACCTCCTGGT
CGB8_chimp   241 ACCTCTTGCCCCCCGAAGGGTTAGTGTCGAGCTCACCCCAGCATCCTACAACCTCCTGGT
CGB7_human   241 ACTTCTCGCCCCCCGAAGGGTTAGTGTCCAGCTCACTCCAGCATCCTACAACCTCCTGGT
CGB7_chimp   241 ACCTCTCGCCCCCCGAGGGGTTAGTGTCAAGCTCACTCCAGCATCCTACAACCTCCTGGT
CGB2_human    98 CCGGCCGGGTTCCCGTGCCGCGTCCAACACCCCTCACTCCCTGTCTCACTCCCCCACGGA
CGB1_human    98 CTGGCCAGGTTCCCGTGCCGCGTCCAACACCCCTCACTCCCTGTCTCACTCCCCCACGGA
CGB1_chimp   100 CCGGCCGGGTTCCCGTGCCGCGTCCAACACCCCTCACTCCCTGTCTCACTCCCCCACGGA
CGB1B_chimp  100 CCGGCCGGGTTCCCGTGCCGCGTCCAACACCCCTCACTCCCTGTCTCACTCCCCCACGGA
```

```
LHB_human      1 --------------------------------------------------------GCAC
LHB_chimp      1 --------------------------------------------------------....
CGB_human    301 GGCCTTGCCGCCCCCACAACCCCGAGGTATAAAGCCAGGTACACGAGGCAGGGGAC....
CGB5_human   301 GGCCTTGCCGCCCCCACAACCCCGAGGTATAAAGCCAGGTACACGAGGCAGGGGAC....
CGB5_chimp   301 GGCCTTGCCGCCCCCACAACCCCGAGCTTTAAAGCCAGGTACACGAGGCAGGGGACA...
CGB8_human   301 GGCCTTGCCGCCCCCACAACCCCGAGGTTTAAAGCCAGGTACACGAGGCAGGGGACA...
CGB8_chimp   301 GGCCTTGCCGCCCCCACAACCCCGAGCTTTAAAGCCAGGTACACGAGGCAGGGGAC....
CGB7_human   301 GGCCTTGACGCCCCCACAAACCCGAGGTATAAAGCCAGGTACACCAGGCAGGGGAC....
CGB7_chimp   301 GGCCTTGACGCCCCCACAAACCAGAGGTATAAAGCCAGGTACACGAGGCAGGGGAC....
CGB2_human   158 GACTCAATTTACTTTCCATGTCCACATC------CCCAGTGCTTGCGGAAGATATCC.G.
CGB1_human   158 GACTCAATTTACTTTCCATGTCCACATT------CCCAGTGCTTGCGGAAGATATCC.G.
CGB1_chimp   160 GACTCAATTTACTTTCCATGTCCACATT------CCCAGTGCTTGTGGAAGATATCC.G.
CGB1B_chimp  160 GACTCAATTTACTTTCCATGTCCACATT------CCCAGTGCTTGCGGAAGATATCC.G.

LHB_human      5 CAAG-GATGGAGATGCTCCAGGGGCTGCTGCTGTTGCTGCTGCTGAGCATGGGCGGGGCA
LHB_chimp      5 ....-................................................T......
CGB_human    361 ....-..........T.........................................A..
CGB5_human   361 ....-..........T.........................................A..
CGB5_chimp   361 ....-..........T...........A.............................A..
CGB8_human   361 ....-..........T.........................................A..
CGB8_chimp   361 ....-..........T.....................................T...A..
CGB7_human   361 ....-..........T.........................................A..
CGB7_chimp   361 ....-..........T.........................................A..
CGB2_human   212 T...A..GA..C...TCAA......................................A..
CGB1_human   212 T...A..GA..C...TCAA..A...................................A..
CGB1_chimp   214 T...A..GA..C...TCAA......................................A..
CGB1B_chimp  214 T...A..GA..C...TCAA.........................T............A..

LHB_human     64 TGGGCATCCAGGGAGCCGCTTCGGCCATGGTGCCACCCCATCAATGCCATCCTGGCTGTC
LHB_chimp     64 .................A...............................C..........
CGB_human    420 ..........A................C......G..............C.........G
CGB5_human   420 ..........A................C......G..............C.........G
CGB5_chimp   420 ......A...A................C......G..............C..........
CGB8_human   420 ..........A................C......G..............C.........G
CGB8_chimp   420 ..........A................C......G..............C..........
CGB7_human   420 ...............AT..........C......G..............C.........G
CGB7_chimp   420 ...............AT..........C......G..............C..........
CGB2_human   272 ..........A................C......G..............C.........G
CGB1_human   272 ..........A................C......G..............C.........G
CGB1_chimp   274 ..........A................C......G..............C..........
CGB1B_chimp  274 ..........A................C......G..............C..........

LHB_human    124 GAGAAGGAGGGCTGCCCAGTGTGCATCACCGTCAACACCACCATCTGTGCCGGCTACTGC
LHB_chimp    124 .................C..........................................
CGB_human    480 .................C..........................................
CGB5_human   480 .................C..........................................
CGB5_chimp   480 .................C..........................................
CGB8_human   480 .................C..........................................
CGB8_chimp   480 .................C..........................................
CGB7_human   480 .................C..........................................
CGB7_chimp   480 .................C...................T......................
CGB2_human   332 .................C..........................................
CGB1_human   332 .................C..........................................
CGB1_chimp   334 .................C..................G.......................
CGB1B_chimp  334 .................C..........................................

LHB_human    184 CCCACCATGATGCGCGTGCTGCAGGCGGTCCTGCCGCCCCTGCCTCAGGTGGTGTGCACC
LHB_chimp    184 ............................................................
CGB_human    540 ..........CC.............G..........G.....................A.
CGB5_human   540 ..........CC.............G..........G.....................A.
CGB5_chimp   540 ..........CC.............G..........G.....................A.
CGB8_human   540 ..........CC.............G..........G.....................A.
CGB8_chimp   540 ..........CC.............G..........G.....................A.
CGB7_human   540 ..........CC.............G..........G.....................A.
CGB7_chimp   540 ..........CC.............G..........G.....................A.
CGB2_human   392 ..........CC.............G..........G.....................A.
CGB1_human   392 ..........CC.............G..........G.....................A.
CGB1_chimp   394 ..........CC.............G..........G.....................A.
CGB1B_chimp  394 ..........CC.............G..........G.....................A.
```

```
LHB_human    244 TACCGTGATGTGCGCTTCGAGTCCATCCGGCTCCCTGGCTGCCCGCGTGGTGTGGACCCC
LHB_chimp    244 ............................................A.....C.........
CGB_human    600 .....C.........................................C..C...A.....
CGB5_human   600 .....C.........................................C..C...A.....
CGB5_chimp   600 .....C.........................................C..C...A.....
CGB8_human   600 .....C.........................................C..C...A.....
CGB8_chimp   600 .....C.........................................C..C...A.....
CGB7_human   600 .....C.........................................C..C...A.....
CGB7_chimp   600 .....C.........................................C..C...A.....
CGB2_human   452 .....C.........................................C..C...A.....
CGB1_human   452 .....C.........................................C..C...A.....
CGB1_chimp   454 .....C........................................AC..C...A.....
CGB1B_chimp  454 .....C.........................................C..C...A.....

LHB_human    304 GTGGTCTCCTTCCCTGTGGCTCTCAGCTGTCGCTGTGGACCCTGCCGCCGCAGCACCTCT
LHB_chimp    304 ....................C.......................................
CGB_human    660 ..........A.G.C...............AA.....C..T................A..
CGB5_human   660 ..........A.G.C...............AA.....C..T................A..
CGB5_chimp   660 ..........A.G.C...............AA.....C..T................A..
CGB8_human   660 ..........A.G.C...............AA.....C..T................A..
CGB8_chimp   660 ..........A.G.C...............AA.....C..T................A..
CGB7_human   660 ..........A.G.C...............AA.....C..T................A..
CGB7_chimp   660 ..........A.G.C...............AA.....C..T................A..
CGB2_human   512 ..........A.G.C...............AA.....C..T................A..
CGB1_human   512 ..........A.G.C...............AA.....C..T................A..
CGB1_chimp   514 ..........A.G.C...............AA.....C..T................A..
CGB1B_chimp  514 ..........A.G.C...............AA.....C..T....T...........A..

LHB_human    364 GACTGTGGGGGTCCCAAAGACCACCCCTTGACCTGTGACCACCCCCAACTCTCAGGCCTC
LHB_chimp    364 ..............................G.............................
CGB_human    720 .....C...........G....................TG......G-..TC....A...
CGB5_human   720 .....C...........G....................TG......G-..TC....A...
CGB5_chimp   720 .....C...........G....................TG......G-..TC........
CGB8_human   720 .....C...........G....................TG......G-..TC....A...
CGB8_chimp   720 .....C...........G....................TG......G-..TC........
CGB7_human   720 .....C...........G....................TG......G-..TC........
CGB7_chimp   720 .....C...........G....................TG......G-..TC........
CGB2_human   572 .....C...........G....................TG......G-..TC........
CGB1_human   572 .....C...........G....................TG......G-..TC....A...
CGB1_chimp   574 .....C...........G....................TG......G-..TC........
CGB1B_chimp  574 .....C...........G....................TG......G-..TC........

LHB_human    424 CTCTTCCTCTAAAGACCCTCCCCGCAGCCTTCCAAGTCCATCCCGACTCCTGGAGCCCT-
LHB_chimp    424 ...........................................................-
CGB_human    779 .........A..G.C........C............C.............C..G.....C
CGB5_human   779 .........A..G.C........C..........................C..G.....C
CGB5_chimp   779 .........A..G.C........C............C.............CC.G.....C
CGB8_human   779 .........A..G.C........C..........................C..G.....C
CGB8_chimp   779 .........A..G.C........C............C.............CC.G.....C
CGB7_human   779 .........A..G.C........C..........................C..G.....C
CGB7_chimp   779 .........A..G.C........C............C......................C
CGB2_human   631 .........A..G.C........C............C.............C..G.....C
CGB1_human   631 .........A..G.C........C.....................T....C..G.....A
CGB1_chimp   633 .........A..G.C........C............C......................C
CGB1B_chimp  633 .........A..G.C........C..........................C..G.....C

LHB_human    483 -GACACCCCGATCCTCCCACAATAAAGGCTTCTCAATCCGCA
LHB_chimp    483 -.........................................
CGB_human    839 G.........................................
CGB5_human   839 G.........................................
CGB5_chimp   839 A.........................................
CGB8_human   839 G.........................................
CGB8_chimp   839 A....T....................................
CGB7_human   839 A.........................................
CGB7_chimp   839 A.........................................
CGB2_human   691 A.........................................
CGB1_human   691 G.........................................
CGB1_chimp   693 A....T................................T...
CGB1B_chimp  693 G.........................................
```
